# Molecular footprints of domestication and improvement in soybean revealed by whole genome re-sequencing

**DOI:** 10.1186/1471-2164-14-579

**Published:** 2013-08-28

**Authors:** Ying-hui Li, Shan-cen Zhao, Jian-xin Ma, Dong Li, Long Yan, Jun Li, Xiao-tian Qi, Xiao-sen Guo, Le Zhang, Wei-ming He, Ru-zhen Chang, Qin-si Liang, Yong Guo, Chen Ye, Xiao-bo Wang, Yong Tao, Rong-xia Guan, Jun-yi Wang, Yu-lin Liu, Long-guo Jin, Xiu-qing Zhang, Zhang-xiong Liu, Li-juan Zhang, Jie Chen, Ke-jing Wang, Rasmus Nielsen, Rui-qiang Li, Peng-yin Chen, Wen-bin Li, Jochen C Reif, Michael Purugganan, Jian Wang, Meng-chen Zhang, Jun Wang, Li-juan Qiu

**Affiliations:** 1 Institute of Crop Science, The National Key Facility for Crop Gene Resources and Genetic Improvement (NFCRI) / Key Lab of Germplasm Utilization (MOA), Chinese Academy of Agricultural Sciences, 100081 Beijing, China; 2 Shenzhen Key Laboratory of Transomics Biotechnologies, BGI-Shenzhen, 518083 Shenzhen, China; 3 Department of Agronomy, Purdue University, 47907, West Lafayette, IN, USA; 4 Institute of Cereal and Oil Crops, Hebei Academy of Agricultural and Forestry Sciences / Shijiazhuang Branch Center of National Center for Soybean Improvement / the Key Laboratory of Crop Genetics and Breeding, 050031 Shijiazhuang, China; 5 Department of Biology, University of Copenhagen, Copenhagen, Denmark; 6 The State Key Laboratory of Plant Cell and Chromosome Engineering, Institute of Genetics and Developmental Biology, Chinese Academy of Sciences, National Centre for Plant Gene Research, Beijing, China; 7 Department of Integrative Biology and Department of Statistics, University of California Berkeley, 94820 Berkeley, CA, USA; 8 Department of Crop, Soil, and Environmental Sciences, University of Arkansas, 72701 Fayetteville, Arkansas, USA; 9 Key Laboratory of Soybean Biology in Chinese Ministry of Education, Northeast Agricultural University, 150030 Harbin, China; 10 State Plant Breeding Institute, University of Hohenheim, Hohenheim, Germany; 11 Department of Biology and Centre for Genomics and Systems Biology, 12 Waverly Place, New York University, 10003 New York, USA

**Keywords:** Artificial selection, Evolution, Genetic diversity, Population genomics, Soybean

## Abstract

**Background:**

Artificial selection played an important role in the origin of modern *Glycine max* cultivars from the wild soybean *Glycine soja*. To elucidate the consequences of artificial selection accompanying the domestication and modern improvement of soybean, 25 new and 30 published whole-genome re-sequencing accessions, which represent wild, domesticated landrace, and Chinese elite soybean populations were analyzed.

**Results:**

A total of 5,102,244 single nucleotide polymorphisms (SNPs) and 707,969 insertion/deletions were identified. Among the SNPs detected, 25.5% were not described previously. We found that artificial selection during domestication led to more pronounced reduction in the genetic diversity of soybean than the switch from landraces to elite cultivars. Only a small proportion (2.99%) of the whole genomic regions appear to be affected by artificial selection for preferred agricultural traits. The selection regions were not distributed randomly or uniformly throughout the genome. Instead, clusters of selection hotspots in certain genomic regions were observed. Moreover, a set of candidate genes (4.38% of the total annotated genes) significantly affected by selection underlying soybean domestication and genetic improvement were identified.

**Conclusions:**

Given the uniqueness of the soybean germplasm sequenced, this study drew a clear picture of human-mediated evolution of the soybean genomes. The genomic resources and information provided by this study would also facilitate the discovery of genes/loci underlying agronomically important traits.

## Background

**Figure 1:**
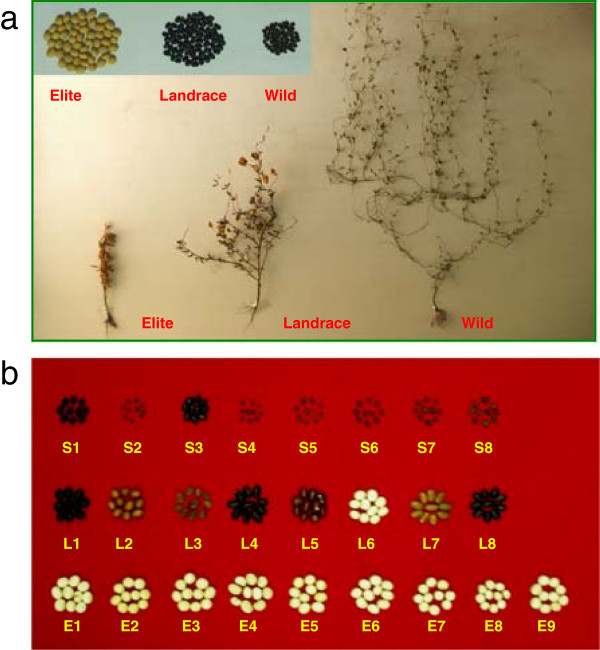
**The photo of 25 re-sequenced soybean accessions.** The photo of **(a)** typical wild, landrace and elite soybean plant and **(b)** seed of 25 re-sequenced soybean accessions.

The modern cultivated soybean [*Glycine max* (L.) Merr.], which contains high protein and oil content, is an important crop worldwide. Soybean was domesticated from its wild progenitor, *Glycine soja* Sieb. & Zucc. ~5,000 years ago [1]. Although the cultivated and wild soybeans show little reproductive isolation and have very similar genomes in both size and content [2], they exhibit substantial morphological difference (Figure 1a). The pre-domesticated wild soybean accessions (*G*. *soja*) have weedy prostrate growth habits and small black seeds, and the domesticated landraces produce smaller plants with less vegetative growth and often are slightly prostrate. In contrast, the elite cultivars developed by modern breeding practices have erect and compact stem architecture with reduced branching, high harvest indices, and high seed yield.

The emergence of cultivated crops from their wild progenitors was achieved primarily by artificial selection for a wide range of desirable traits to meet human needs [3,4]. Although domestication traits were often controlled by a relatively small number of genes, including major quantitative trait loci (QTL) and/or Mendelian loci, selection for such traits would have resulted in a progressive reduction of genetic diversity throughout the genome [3]. Genetic diversity was further reduced following domestication by modern breeding practices [5]. The genetic bottlenecks associated with the domestication and genetic improvement of soybean had been illustrated by analysis of 111 fragments from 102 genes [6]. To date, several agronomically important genes including the *Dt1* locus controlling soybean stem growth habit and *E* genes (*E1*-*E4*) controlling flowering time have been cloned by homology-based or map-based approaches [7-12]. Nevertheless, little is known about how genetic diversity across the whole genome of soybean was shaped by domestication.

The availability of the soybean genome sequence [13] and high throughput sequencing technologies provides an unprecedented opportunity to track the evolutionary history of domesticated soybean, and to dissect the genetic bases for soybean domestication and varietal diversification. Recently, for example, 31 soybean accessions, representing wild and cultivated gene pools, had been re-sequenced and analyzed [14]. This study shed light on the nature and extent of genetic differentiation between wild and cultivated soybean species. Nevertheless, no information about landraces – the bridge between wild soybean (domestication) and elite cultivars (improvement) was provided. Investigations of the loss and recovery of genetic diversity in the course of soybean domestication and breeding would provide guidelines and strategies for utilization of landraces and/or wild accessions for soybean enhancement. Moreover, comparative genomics analyses among wild, landrace, and elite soybeans would identify genes under selection. The knowledge obtained from these analyses will facilitate the introgression of beneficial alleles from wild soybean and landraces to elite cultivars.

In this study, we re-sequenced 25 diverse soybean accessions, which represent three distinct gene pools: the pre-domesticated annual wild progenitor species (*G*. *soja*), domesticated local landraces (*G*. *max*), and modern elite cultivars (*G*. *max*). To achieve a more comprehensive analysis, we integrated these re-sequencing data with the re-sequencing data previously generated from 14 wild and 17 cultivated soybean genomes [14]. Our study not only elucidated the trends of molecular diversity, but also identified distinct footprints in the soybean genomes associated with artificial selection during soybean domestication and elite cultivar development.

## Results and discussion

### High quality sequence data was generated for 25 diverse soybean accessions

We used 25 diverse soybean accessions in this study: eight wild soybeans, eight landraces, and nine modern elite cultivars. To maximally represent the genetic diversity and wide geographic distribution, this panel of accessions was selected based on intensive molecular and phenotypic characterization, which reflect the major operational taxonomic units (OTUs) of soybeans in China [15] (Figure 1b, Additional file 1 and Additional file 2). Using the genome-wide re-sequencing approach, a total of 1.356 billion high-quality paired-end reads (93.55 Gb) were generated (Additional file 3), covering 98.2% of the genome sequences (c.v., Williams 82, Glyma1.01). To overcome potential ambiguity caused by sample size and low-pass sequencing in detecting SNPs [16], we downloaded the 31 soybean re-sequencing data through the NCBI Short Read Archive (accession number: SRA020131). After calibrating the SNP calling quality by all the 55 accessions (except the neutron-mutated line C16 from NCBI) and discarding singletons and most doubletons according to rigorous filtering criteria [17,18], we identified 5,102,244 high quality SNPs in our sequenced accessions (Additional file 4, http://www.ncbi.nlm.nih.gov/SNP/snp_viewTable.cgi?handle=NFCRI_MOA_CAAS), which was slightly lower than that discovered previously in the 31 soybean accessions (http://public.genomics.org.cn/BGI/soybean_resequencing/). Among these, 25.5% (1,299,265) SNPs were newly reported here. Additionally, we identified 701,792 small (<5 bp) insertion/deletions (InDels), which provide useful markers for mapping genes, and 6,177 large deletions (>200 bp), with a mean length of 3,615 bp. We validated 106 SNPs from ten randomly selected genes using the Sanger method, and the accuracy of SNP calling reached 97.3%, suggesting that potential miscalling of SNPs in this study was minimal.

### Bayesian clustering revealed introgression of the wild into the cultivated soybeans

**Figure 2:**
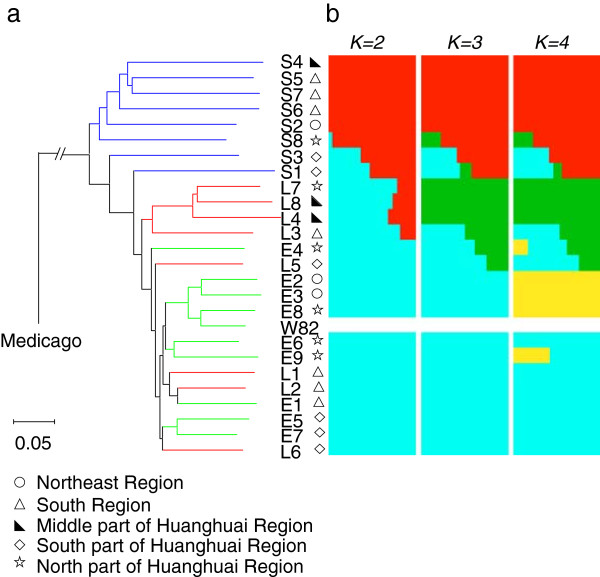
**Phylogenetic tree and population structure of 25 re-sequenced soybean accessions. a**, Neighbor-joining tree of soybean accessions. Northeast region. South region. Middle part of Huanghuai region. North part of Huanghuai region. South part of Huanghuai region. W82, Williams 82. **b**, Population structure inferred by ADMIXTURE. Each accession shown as a vertical line partitioned into *K* colored components represents inferred membership in *K* genetic clusters. Blue, wild lines; red, landrace lines; green, elite cultivars.

Phylogenetic relationships of the 25 accessions and Williams 82 [13] were established using another legume model, *Medicago truncatula*[19] as an out-group. The cultivated and the wild soybeans were separated into two groups (Figure 2, Additional file 5), suggesting that the domestication event promoted the genetic differentiation within the subgenera *Soja*. Within the cultivated accessions, the lines L3, L4, L7 and L8 were separated from the other cultivated accessions.

The Bayesian clustering approach revealed different degrees of introgression between the cultivated and the wild groups (Figure 2b). It is particularly interesting that the four landraces (L3, L4, L7 and L8) with mosaic pattern at *K* = 2 were found to have at least one of the wild traits, such as small seed size, dark seed-coat color, and seed-coat bloom (typical wild phenotypes). In contrast, two wild accessions (S1 and S3) showing admixture carrying one of correspopnding typical cultivated phenotypes (Additional file 1). A recent study revealed that the *Oryza sativa* indica, a cultivated rice subspecies, was developed from crosses between the other cultivated rice subspecies, *O*. *sativa* japonica and its wild progenitor *O*. *rufipogon*[18] suggesting that introgression between the wild and cultivated species and re-selection for desirable agronomic traits may be a common process for crop domestication. Further re-sequencing of larger populations of representative wild and cultivated soybeans such as core collections would allow full elucidation of such evolutionary events occurred during soybean domestication.

Within the cultivated soybean group, the landraces were not separated from elite cultivars distinctly (Figure 2a, Additional file 5). Instead, individuals from the same geographical region tended to cluster together, which reflected isolation by distance during evolution and/or parallel selections in similar ecological habitats accompanied by gene flow.

### Genome diversity was more impacted by domestication than by genetic improvement

**Figure 3:**
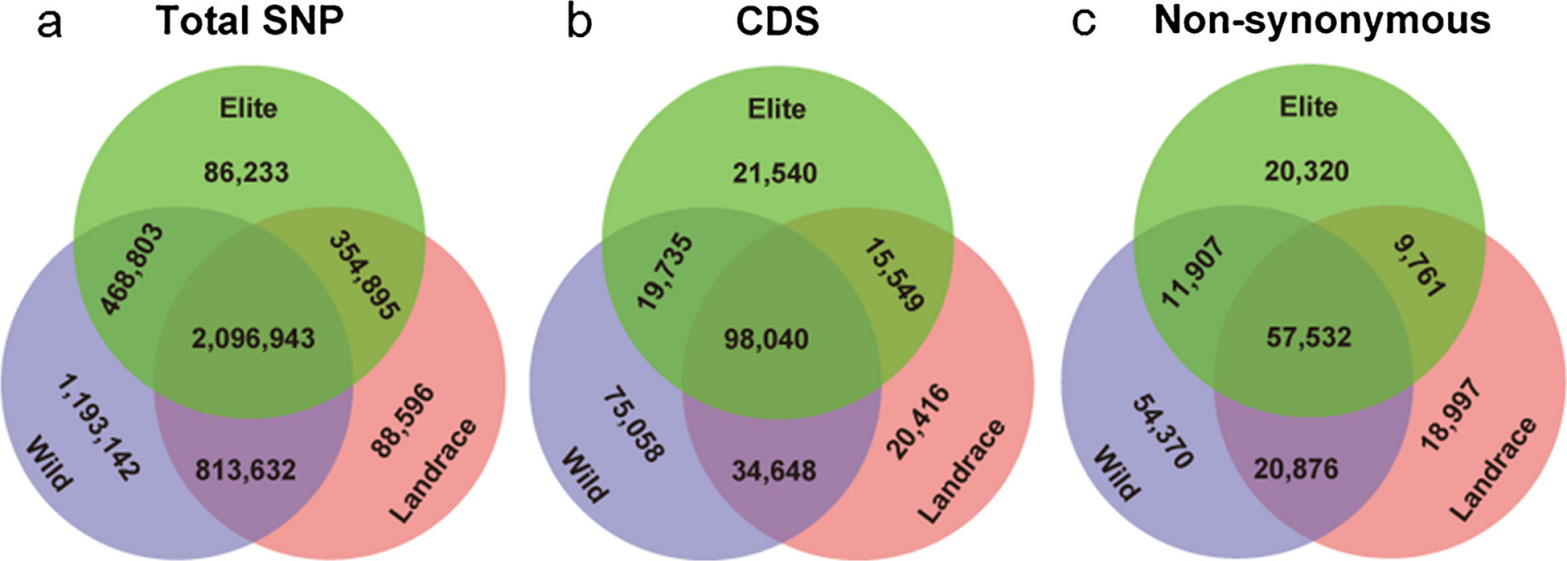
Overlap of (a) total SNPs, (b) SNPs in CDS region, and (c) non-synonymous SNPs in three soybean gene pools (wild, landrace and elite cultivar).

Number of SNPs as well as nucleotide diversity substantially decreased throughout the domestication process from the wild to the cultivated soybeans, which was consistent with previous studies [6,15,20,21]. Our data revealed that 1,661,945 SNPs in wild soybean were not polymorphic in the landraces (Figure 3). Of these SNPs, 5.7% (94,793) were located in the CDS regions of genic sequences and 4.0% (66,637) were non-synonymous sites. In addition, we observed a reduction of 31% and 26% of genetic diversity from the wild soybeans to landraces, as measured by *θ*_*π*_ and *θ*_*w*_ respectively [22] (Additional file 6). These observations contrasted with a previous study, which reported a reduction of nucleotide diversity from *G*. *soja* to landraces at 34% and 51%, measured by *θ*_*π*_ and *θ*_*w*_, respectively [6]. Different samples and different sets of genes were investigated in these two studies, which might explain the different levels of reduction of genetic diversity detected in the two studies.

It is hypothesized that modern plant breeding reduces genetic diversity in elite cultivars, consequently jeopardizing future crop improvement [5]. Although this conception appears to be true for most crop species, our data showed limited effects of breeding on reduction of genetic diversity. We found that the elite gene pool harbored a high proportion of the genetic diversity (83.8% for *θ*_*π*_ and 87.8% for *θ*_*w*_) presented in the landraces (Additional file 6), contrasting with a previous study by Hyten et al. [6], which demonstrated that the elite cultivars retained 78% (*θ*_*π*_) and 72% (*θ*_*w*_) of the diversity present in the landraces. This difference may indeed reflect the relative levels of genetic diversity of the two sets of elite soybean cultivars investigated in both studies.

The number of fixed SNPs from landraces to elite cultivars (899,865) was only half (54%) of the number of fixed SNPs during domestication (Figure 3, Additional file 4). Similar patterns were observed when only one gene component, such as intron, CDS, or UTR, was analyzed (Figure 3, Additional file 4). Together, these observations indicated that the impact of intensive selection by modern soybean breeding on reduction of genetic diversity was less severe than that of selection by the domestication process, suggesting that the wild soybean gene pool was the major reservoir that retained genes/alleles lost during domestication and modern breeding practice. We would like to point out that this interpretation would be largely affected by the genetic base of ancestral landraces that were used for the development of elite cultivars investigated in this study. Nevertheless, similar observations were also observed in maize. A recent study by Hufford et al. demonstrated a remarkably weak genome-wide genetic bottleneck by mordern maize breeding [23].

### Decrease in the haplotype diversity during domestication

The extent of linkage disequilibrium can be interpreted as a measurement of haplotype diversity in a population. We observed a drastic increase in linkage disequilibrium (LD) across the whole genome from wild to landraces and elite cultivars (Additional file 7) pointing to a severe loss of haplotype diversity. This observation reflects the genetic bottleneck during domestication, which reduced the genetic diversity throughout the genome by eliminating some recombinant lineages. It is likely that the lower level of outcrossing rate of the cultivated soybean relative to the wild soybean [24] contributed to an increase in LD in the former. By contrast, the LD pattern of the landraces differed only slightly from modern elite cultivars (Additional file 7). As a result, the resolution of genome-wide association mapping for panels of landraces or elite cultivars was much lower than that for the wild soybeans. We also observed a large variation in extent of LD among different chromosomes (Additional file 7), suggesting that molecular markers designed for genotyping strategies should be specific to genomic regions in association mapping analyses. For example, relatively low density of markers is needed for the regions with relatively extensive LD.

### Footprints of domestication in the soybean genome

**Figure 4:**
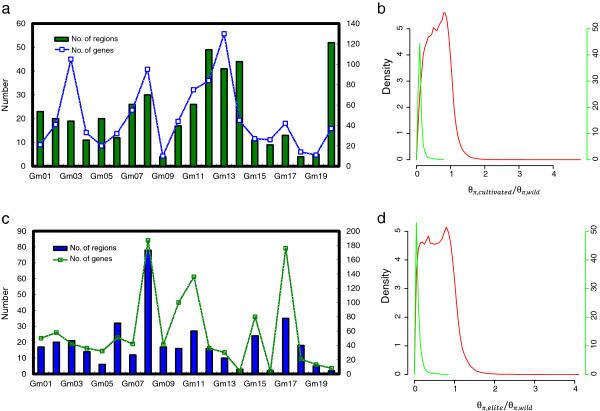
**Detection of candidate genome region and genes underwent selection during domestication and genetic improvement.** The distribution of domestication **(a)** and genetic improvement **(c)** regions and genes on the soybean chromosomes. Rectangular bar, number of regions; line, number of genes. Polymorphism distribution between cultivated (landrace plus elite) and wild populations **(b)**, as well as between elite and wild groups **(d)** in candidate region (green line) versus the whole genome (red line).

The loss of genetic diversity during domestication and genetic improvement is likely due to the fixation and sweep of alleles caused by population bottlenecks or artificial selection. We scanned a combined dataset of 55 accessions to identify genome-wide signatures of artificial selection following a bottom-up genetic approach [25]. To detect the reduction of genetic diversity caused by domestication, we employed a sliding window strategy to estimate *θ*_*π*_[26] and Tajima’s D [27]. The regions that showed significantly lower *θ*_π_ in landrace relative to the wild group (Z test, *P* < 0.05) and significantly lower Tajima’s D (Z test, *P* < 0.05) in landraces relative to the wild group were considered as putative domestication-related regions. This approach has been used to study domestication event in silkworms [17] and rice [18]. The genome scan revealed that only 1.47% of the whole genome (950 M), comprising 394 regions distributed on individual chromosomes (Figure 4a), appeared to have been affected by selection during domestication. The length of these regions ranged from 20 kbp to 280 kbp and the polymorphism levels of these regions relative to the whole genome were relatively low (Figure 4b). A total of 928 genes were located in the regions with footprints of artificial selection, accounting for 2.0% of the 46,430 predicted genes in the cultivated soybean genome [13].

It was reported that some QTLs controlling mesdotication-related traits located in syntenic regions among different species [28]. We found that some candidate genes related with soybean domestication detected in this study had homologs, which were also affected by artificial selection in other crops, such as rice and sunflower. For example, *Glyma03g35520*.*1*, which is probably involved in the carbohydrate metabolism pathway, was found to be an orthologous gene of *Grain Incomplete Filling 1* (*GIF1*), a domestication gene identified in rice [29]. *GIF1* encodes a cell-wall invertase that regulates sugar levels for cell division and growth during grain development, resulting in higher seed weight – an important trait for rice domestication. In addition, we found a strong selection signal for *Glyma03g35250*.*1*, an orthologous gene of *Terminal Flower 1* (*TFL1*), which experienced selective sweeps in the domestication of sunflower [30]. As the closest paralogous gene of *Glyma03g35250*.*1* in soybean, *GmTfl1* (*Glyma19g37890*.*1*) was identified to control the agronomically important trait indeterminacy (*Dt1*/*Dt1*), which is associated with soybean domestication and varietal differentiation [7,8]. Nucleotide diversity analysis of 20 wild and 89 cultivated soybeans detected five SNPs in the wild population, but none of them were found in the cultivated population, suggesting that *Glyma03g35250*.*1* had experienced artificial selection [7].

### Footprints of intensive breeding in the soybean genome

Population branch statistics (PBS) is an effective method to detect signatures of recent natural selection [31]. Taking wild soybeans as a control in the PBS approach, we found that 306 regions were associated with significant signs (*P* < 0.001) of artificial selection by the modern breeding practice (Figure 4c, Figure 4d). These regions spanned a total of 14,462 kbp in length, corresponding to 1.52% of the whole genome (950 M). Of these 306 regions, 271 were found to harbor a total of 1,106 genes showing signatures of selection, which account for 2.4% of all the genes located in these 271 regions [13]. No genes were annotated in the remaining 35 regions.

The black seed-coat progressively changed to various colors during domestication, with positive selection for yellow in the following improvement. Multiple alleles at the *I* locus were found to be associated with an unusual cluster of five chalcone synthase genes (*CHS1*, *CHS3*, *CHS4*, *CHS5*, and *CHS9*) that controlled the distribution of seed-coat color by inhibiting coloration over the entire seed coat [32,33]. In this study, three (*Glyma08g11520*.*1*, *Glyma08g11530*.*1 and Glyma08g11610*.*1*) of these five candidate *CHS* genes showed strong selection signals.

The evolution of flowering time was crucial for developing cultivars adapted to a wider geographical regions [34,35]. We found that two genes related to flowering time, *GmCRY1a* (*Glyma04g11010*.*1*) and *Glyma10g42090*.*1*, exhibited selection signals. *GmCRY1a* was a major regulator of photoperiodic flowering in soybean and had an important role in determining latitudinal distribution of soybean [36] while *Glyma10g42090*.1 was a homologous gene of CONSTANS (*CO*), which was found to encod a key protein involved in photoperiod sensing in *Arabidopsis*[37].

In total, 4.38% of the annotated genes were impacted by artificial selection for agricultural traits. Polymorphism levels in the detected selection regions were relatively low compared to that of the whole genome (Figure 4b, Figure 4d). The percentage of candidate genes impacted by artificial selection was similar to that was estimated in maize (about 2% to 4%) [23,38]. However, this was slightly lower than that reported (~5%) by Lam *et al*. [14] probably due to the sampling effects and different analytical methods employed. Only two regions located on Gm03 and Gm15 showed selection signatures for both domestication and subsequent modern breeding practice. The selected genes appeared to be distributed in clusters in certain genomic regions (Additional file 8), similar to the distribution pattern of domestication-related QTLs defined by QTL mapping [28]. The domestication and improvement related genes were clustered into 386 gene families by OrthoMCL [39]. Of these 386 genes, 230 were shared by both processes.

**Figure 5:**
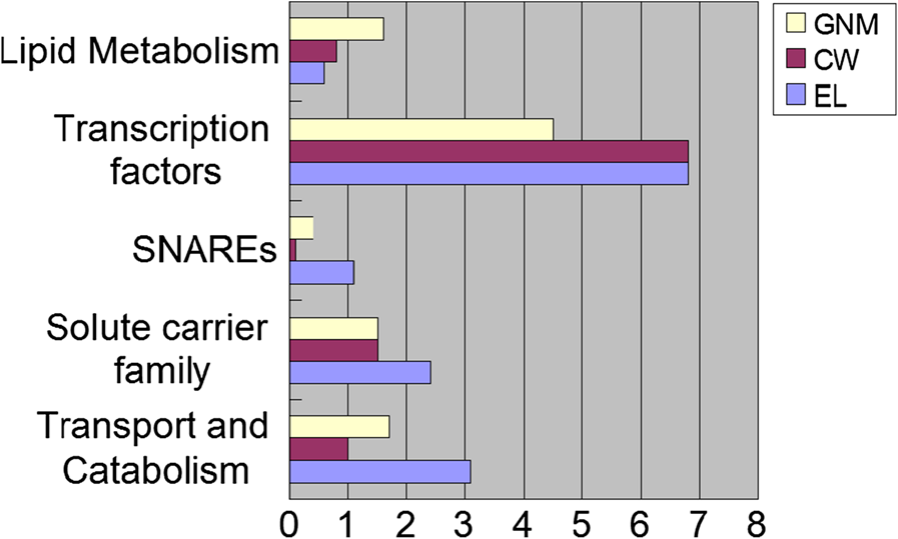
**Accumulation of domestication and improvement genes in different pathways of KEGG (χ**^**2 **^**test, *P*** **< 0.05).** CW: domestication genes; EL: improvement genes; GNM: genome wide genes.

Using the KEGG (Kyoto Encyclopedia of Genes and Genomes) [40] database, potential functions of the selected genes were predicted. We found that the selected genes were significantly (χ^2^ test, *P* < 0.05) involved in lipid metabolism, transcription factors, SNAREs (soluble N-ethyl-maleimide sensitive factor attachment protein receptor), solute carrier family, and transport and catabolism (Figure 5). Growing demand for vegetable oil is a paramount objective of soybean domestication and genetic improvement, which has focused selection toward cultivars with high accumulation of lipids [41,42]. A high frequency of selected genes involved in lipid metabolism was also observed during both processes (Additional file 9). This indicates that continuous artificial selection had occurred in the pursuit of preferred-quality soybean seed. These preliminary data would allow us to prioritize further analyses with an emphasis on understanding of the biological functions of selected genes.

Similar to described in maize [43], transcription factors were enriched in the candidate genes with selection signaturs, suggesting that these regulatory genes had been the major target of selection. Of the 19 domestication-related genes identified in any plant species to date, [44-51], 12 were transcription factor genes [45,46,48]. These genes were responsible for major morphology differentiation between cultivated crops and their progenitors, such as branch (*tb1*) and glume architecture (*tga1*) in maize [52,53], seed size (*fw2*.*2*) and style length (*Style2*.*1*) in tomato [54,55], seed color (*R* and *Q*) genes in wheat [56,57], six-rowed spike (*vsr1*) in barley [51], seed shattering (*qSH1*, *sh4* and *APETALA2*) [46,58,59] in rice and in cereal including sorghum, rice and maize (*Sh1*) [50], fruit opening and seed dispersal (*RPL*) in Brassicaceae [45]. A recent study accounted well for this observation which observed that the regulatory genes with stronger regulatory action on the other genes are the targets of selection within the complex regulatory networks inferred from a simulation study using a matrix model [44].

### Discovering genes with an integrated QTL mapping and re-sequencing approach

Although genomic regions and genes, most likely affected by artificial selection, had been identified, the functions and phenotypes of these genes remained elusive [25]. To validate footprints of selection during domestication and genetic improvement, we compared the genomic regions with previously mapped QTLs, which were identified from interspecific populations and intraspecific populations developed by crossing landrace and cultivar, respectively (Additional file 10). A total of 21 candidate domestication regions including 60 genes were covered by the mapped domestication QTLs or their adjacent regions [60-64]. Important agronomic traits included yield, plant height, lodging, maturity time, seed weight, seed hardness, seed-coat color, and flower color. And a total of 20 candidate improvement regions including 106 genes were covered by improvement QTLs or their adjacent regions [65-67].

**Figure 6:**
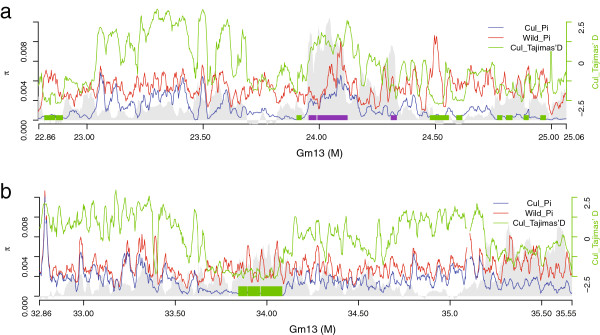
**The gene diversity of genomic regions of seed size (a) and seed coat blooming (b) on chromosome 13.** Top, Tajima’s D (green line); LE PBS (gray shading). Genomic diversity of wild group (red dotted line) and cultivated group (blue dotted line) displayed by π (pi) are plotted using 500 kbp sliding windows. The square frames along the chromosome indicate regions selected during domestication (green) and genetic improvement (purple).

In addition, the integration of selection regions identified using population genetic analysis method with QTLs region identified using an bi-parents populations may be a useful approach to narrow down the broad QTLs [68]. We conducted a linkage mapping study in an interspecific F_2:3_ population consisting of two of the parents included in our survey and searched for QTL for seed size, one of the most prevailing domestication phenotypes (Figure 1b). Among the detected QTL we observed one at linkage map of LG F (Gm13), which accounted for 15.1% of seed size variation. The genomic distance between the two flanking markers Satt425 and Satt114 was 4.8 Mb (from 22,874,022 bp to 27,718,828 bp of Gm13). The selection signals were further identified in eight internal regions (258 kbp) using 500 kbp sliding windows in the QTL (Figure 6a). Within the narrowed regions, 17 genes were potentially responsible for seed size variation. Four regions (190 kbp) were identified as footprints of intensive breeding in this QTL region and a seed size QTL was also discovered nearby using an intraspecific cultivated soybean population [69], indicating that artificial selection occurs continuously in or near the QTL in the pursuit of higher production. We further identified selection signals within another QTL on Gm13, which is responsible for the typical soybean domestication trait, seed blooming (*B1*) [70] (Additional file 9). In 2.7 Mb of this QTL region, three nearby candidate domestication regions consist of 234 kbp DNA were identified (Figure 6b). This approach offers potential application for cloning candidate genes underlying the domestication traits of soybean as well as other crops.

## Conclusions

Soybean has undergone a series of selections over time, natural or artificial, intentional or unintentional, leading to the decrease in genetic diversity from the wild progenitor to landraces and from landraces to the modern elite cultivars. We reported that whole genome re-sequencing analysis enhanced our understanding of genetic diversity in wild and cultivated soybeans, and unraveled the processes how this important legume species was domesticated. In present study, the strength of genetic bottlenecks caused by domestication and modern breeding were demonstrated. The continuing reduction of genetic diversity in the cultivated soybean has become a bottleneck for improvement of soybean cultivars. We currently have unprecedented opportunities to exploit genetic diversity in the wild soybean and landraces for sustainable enhancement of soybeans.

A set of candidate genes/regions were identified, significantly impacted by selection, for constructing preferred traits underlying soybean domestication and genetic improvement. Comparison of candidate domestication and crop improvement-related genes with previous QTL mapping results, as well as their homologs, provides information on potential function(s) of genes under artificial selection. In particular, we found genes related to seed-coat color, growth habit, flowering time and seed size, which had been confirmed as continuously changing from wild soybeans to landraces and then elite cultivars. Further analysis is required to identify how variation in these candidate genes affect phenotypes using QTL mapping e.g. in maize [71], association mapping e.g. in barley [72], gene expression assays e.g. in sunflower [30], and gene-knock-out methods [43]. Our findings, however, promote development of more efficient approaches to identify the genes underlying domestication-related traits. This study also contribut0065 to construct a large-scale soybean haplotype map and discover important trait related genes using genome-wide association studies. Our understanding of the nature of genetic diversity in wild and cultivated soybeans, and the impact of domestication and breeding on genome diversity, will aid future breeding of elite cultivars to improve soybean production and meet the increasing worldwide demands for feed, vegetable oil, soyfood and biofuels.

## Methods

### Sample collection for whole genome sequencing

We selected eight landraces and nine elite cultivars/lines from the Chinese soybean mini-core collection [73,74] and five *G*. *soja* accessions on the basis of geographic distribution and genotypic diversity. These represent all major operational taxonomic units (OTUs) of the Chinese soybean germplasm and 98.8% of gene diversity [15]. To ensure balanced geographic distribution, three annual wild soybeans were collected. Most of the elite cultivars/lines are widely cultivated in China. Our panel of 25 accessions originates from the Northeast region, Huanghuai region (including north, middle and south parts) and South region of China, from 24.1 to 46.4 °N and from 102.4 to 126.6 °E, which represent the four major soybean cultivation areas in China [75]. These accessions were obtained from the Chinese National Soybean GenBank.

We also integrated the 30 (except C16, a neutron-mutated line) soybean re-sequencing data of Lam *et al*., from the NCBI Short Read Archive (accession number: SRA020131) [14], in SNP calling procedures and screening of selection regions. The information of these 30 accessions can be found in the website: http://wildsoydb.org/strains/soybean (personnal communication with Prof. H.M. Lam at The Chinese University of Hong King).

### QTL mapping population

A total of 85 F_2_ generation progenies were derived from the cross between the *G*. *max* cultivar E9 (Jidou12) and a *G*. *soja* accession S8 (ZYD02738). All of the F_2_ plants were selfed to develop F_2:3_ using pedigree method. Field trials were conducted at the sandy soil in the Dishang Experimental Station of the Institute of Cereal and Oil Crops in Hebei, China (114.29°E, 38.04°N) in 2009. The F_2:3_ population and the parents were grown in a randomized complete block design with three replications. Each plot consisted of one row with 1.0 m wide and 3.0 m long with a space of 30 cm between two plants. Standard agronomic practice including were followed to maintain a weed-free field. Seven plants in each row were used to measure 100-seed weights and extract DNA.

### Whole genome sequencing and alignment

For each sample, total genomic DNA was extracted from fresh leaves of dark-grown plants at the first trifoliolate stage using the DNeasy Plant Mini Kit (QIAGEN). The DNA library for sequencing was prepared following the manufacturer’s instructions (Illumina). Short reads were derived from the raw image files by applying Illumina base-calling Pipeline (SolexaPipeline 1.3.4). These were subsequently aligned onto the soybean reference genome (*Glycine max* var. Williams 82, http://www.jgi.doe.gov) [13] using SOAP2 [76] with parameters: -a –b –D –o −2 –u –m –x –v –l 32 –s 40. A maximum of five mismatches were allowed for the 75 bp read and three for the 44 bp read. The alignment results were classified into three types: unique mapped, repeat mapped and unmapped reads. PCR duplication reads during sequencing, which affect the sequencing depth and variation detection, were excluded by an in-house script.

### SNP/InDel calling and validation

Both the Bayesian theory and the maximum likelihood estimation method were applied to population SNP calling. Genotype likelihood of each genomic site for each line was calculated by SOAPsnp [16], which considers four main attributes: 1) o_k_, observed allele type; 2) q_k_, sequencing quality; 3) c_k_, read coordinate; and 4) t_k_, the t_k_-th observation of the same allele from reads with the same mapping location. For each assumed genotype H, the likelihood P(d_k_|H) = P((o_k_, q_k_, c_k_)|H) = P((o_k_, c_k_)|(H, q_k_)) * P(q_k_|H). Here, we used d_k_ to represent the attributes, o_k_, q_k_, c_k_ and t_k_.

All individual likelihood results were integrated to generate pseudo-chromosomes for every site of all samples by maximum likelihood estimation. Finally, for each site, certain criteria were used to improve accuracy: 1) the depth >20 && <160; 2) the copy number < =1.5; 3) the quality score given by SOAPsnp >20; and 4) examination of each heterozygous site by rank sum test based on the quality values of mapped bases. To validate our results, we randomly selected ten genes containing 106 SNP sites for PCR-Sanger sequencing using the AB 3730XL.

Small insertion and deletion (InDel) calling was also processed using a previously described method [17]. Three steps were followed to call InDels: 1) reads were realigned with SOAP2 allowing gaps; 2) considering the supporting reads for each site, at least one individual InDel existed in the population; 3) allotted InDels back to each individual.

### Population structure and phylogenetic analysis

We constructed a phylogenetic tree by a neighbor-joining method in the software PHYLIP (version 3.68) [77]. A total of 1,000 replicates generated the bootstrap values. We then used a likelihood-based method with the program ADMIXTURE [78] to investigate the ancestry information of soybean genotypes, using PLINK [79] for genotype quality control. Using the principal component analysis (PCA), the population subdivision pattern was then inferred [80].

### Linkage disequilibrium (LD)

To evaluate the LD pattern in wild, landrace, and elite soybean groups, we estimated the squared allele frequency correlation (*r*^2^) of alleles using Haploview 1.4 [81], setting the parameters as: -maxdistance 1000 -dprime -minGeno 0.6 -minMAF 0.1 –hwcutoff 0. The LD decay graphs were plotted using R script for each population and for individual chromosomes.

### Genome diversity and selection

To estimate the genetic diversity, we calculated the average pairwise divergence within a population (*θ*_*π*_) and the Watterson’s estimator (*θ*_*w*_) [22] for the whole genome of wild, landrace, and elite populations. The 20 kbp sliding window with 2 kbp step-size along the genome was used to estimate these two parameters with an in-house PERL script.

To identify genomic footprints of artificial selection, we used an outlier approach looking for genetic bottlenecks. We applied two methods to identify candidate selection regions in the genome. First, using a 20 kbp sliding window (2 kbp step-size), we compared sequence diversity between wild annual and cultivated soybean groups. For each window, we estimated *θ*_*π*_ and Tajima’s D. Those regions that had significantly low *θ*_*π*_.cultivated/*θ*_*π*_.wild and low D values (Z test, *P* < 0.05 for both) in cultivars were putative selected regions. Additionally, the pair-wise nucleotide diversity and Tajima’s D were also applied to evaluate genome diversity of different populations. Second, we chose the population branch statistic on the basis of *F*_*st*_[31] to infer the selective footprints from landrace to elite cultivar. This approach had been shown to be effective in identifying recent artificial selection [17] considering the very short divergence time between landrace and elite cultivar.

### QTL mapping

Ten simple sequence repeats (SSRs) from linkage group F (Gm13) (http://www.ars.usda.gov) were used to genotype the F_2:3_ population derived from the E9 (Jidou12) × S8 (ZYD02738) cross. QTL (LOD > 2.5) were detected by single marker analysis and interval composite interval mapping (ICIM), implemented by QTL IciMapping v3.0 (http://www.isbreeding.net). For ICIM, the scanning step-size was set at 1, and the probabilities for markers moving into and out of the model were set at 0.05 and 0.10, respectively.

### Data availability

All sequence read data was deposited in Sequence Read Archive (SRA) under accession number SRP015830. The SNPs were also available in Database of Short Genetic Variations (dbSNP) with batch id 1058942.

## Abbreviations

CHS: Chalcone synthase; CO: CONSTANS; Gb: Gigabase; G. max: *Glycine max* (L.) Merr.; G. soja: *Glycine soja* Sieb. & Zucc.; GIF1: Grain incomplete filling 1; ICIM: Interval composite interval mapping; InDels: Insertion/deletions; KEGG: Kyoto encyclopedia of genes and genomes; LD: Linkage disequilibrium; OTUs: Operational taxonomic units; PBS: Population branch statistics; PCA: Principal component analysis; QTL: Quantitative trait loci; SNAREs: Soluble n-ethyl-maleimide sensitive factor attachment protein receptor; SNPs: Single nucleotide polymorphisms; SSRs: Simple sequence repeats; TFL1: *Terminal flower i.*

## Competing interests

The authors declare that they have no competing interests.

## Authors’ contributions

L-J Qiu, J Wang, R-Z Chang and J Wang designed the research; Y-H Li, L Yan, X-T Qi, L Zhang, Y Guo, X-B Wang, R-X Guan, Y-L Liu, K-J Wang, L-G Jin, Z-X Liu, L-J Zhang, X-Q Zhang and J-X Li performed the research. Y-H Li, S-C Zhao, D Li, J-Y Wang, J Li, X-S Guo, W-M He, Q-S Liang, C Ye, J Chen, W-B Li, M-C Zhang, Y Tao, and R Nielsen analyzed the data. Y-H Li, S-C Zhao, D Li, J-X Ma, P-Y Chen, R-Q Li, J C Reif, M Purugganan and L-J Qiu wrote the manuscript. All authors read and approved the final manuscript.

## Supplementary Material

Additional file 1 The geographical, ecotype and domestication-related traits information of 25 soybean accessions worldwide. Click here for file

Additional file 2 **The geographic distributions of 25 soybean accessions. ***Glycine soja* is represented by the green hollow circle, landrace is the red hollow rhombus and elite cultivar is the blue triangle. The sky-blue lines divide China into four regions: Northeast, North, Huanghuai and South regions. The black lines represent the Yellow and Yangtze rivers. Click here for file

Additional file 3 Sequencing of 25 soybean accessions represented wild, landrace and elite gene pools. Click here for file

Additional file 4 SNP distribution in the wild, landrace and elite soybean gene pools. Click here for file

Additional file 5 Principal component analysis (PCA) of 25 soybean accessions from wild, landrace and elite cultivar gene pools. Click here for file

Additional file 6 **Pairwise nucleotide diversity (*θ***_***w ***_**and *θ***_***π***_**) on the genome-wide level.** Click here for file

Additional file 7 **LD decay determined by squared correlation coefficient of allele frequencies (*r***^***2***^**) in against distance among three soybean gene pools on whole genome level (a) and at each chromosome (b).** Click here for file

Additional file 8 **The diversity pattern of artificial selection regions during domestication and genetic improvement based on Fst, Tajima’s D, π, or PBS’s analysis (between Landraces and elite cultivars).** The square frames along the chromosome indicate regions selected during domestication (green) and genetic improvement (purple). Click here for file

Additional file 9 **Pathway analysis for domestication and improvement genes by KEGG.** The pathway marked with “&” was deduced from KEGG PATHWAY database and marked with “#” deduced from KEGG BRITE database. Click here for file

Additional file 10 Domestication regions and genes covered by or near reported QTLs for important domestication traits. Click here for file
