# Supplementary figures and images for: Molecular footprints of domestication and improvement in soybean revealed by whole genome re-sequencing
Source: BMC Genomics. 2013 Aug 28;14:579. doi: 10.1186/1471-2164-14-579 (PMC3844514; doi:10.1186/1471-2164-14-579)

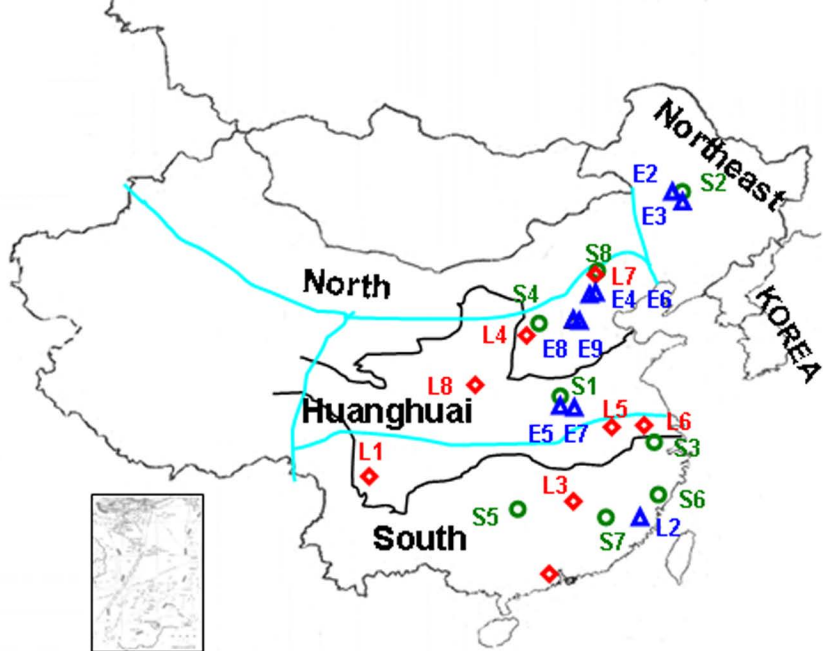

Spratly Islands

Supplement: Additional file 2 — The geographic distributions of 25 soybean accessions. Glycine soja is represented by the green hollow circle, landrace is the red hollow rhombus and elite cultivar is the blue triangle. The sky-blue lines divide China into four regions: Northeast, North, Huanghuai and South regions. The black lines represent the Yellow and Yangtze rivers. [file 1471-2164-14-579-S2.pdf]

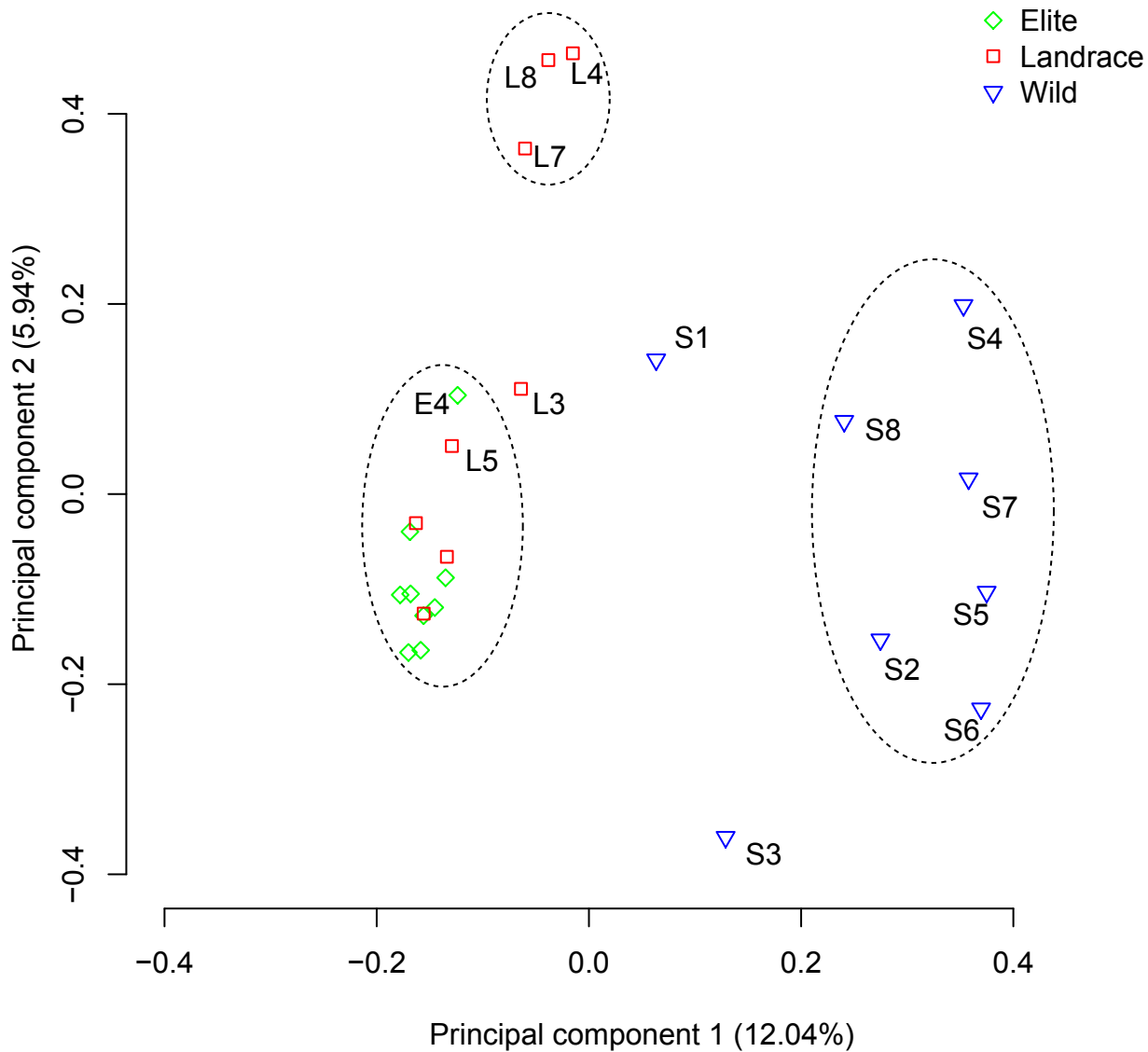

Supplement: Additional file 5 — Principal component analysis (PCA) of 25 soybean accessions from wild, landrace and elite cultivar gene pools. [file 1471-2164-14-579-S5.pdf]

a.

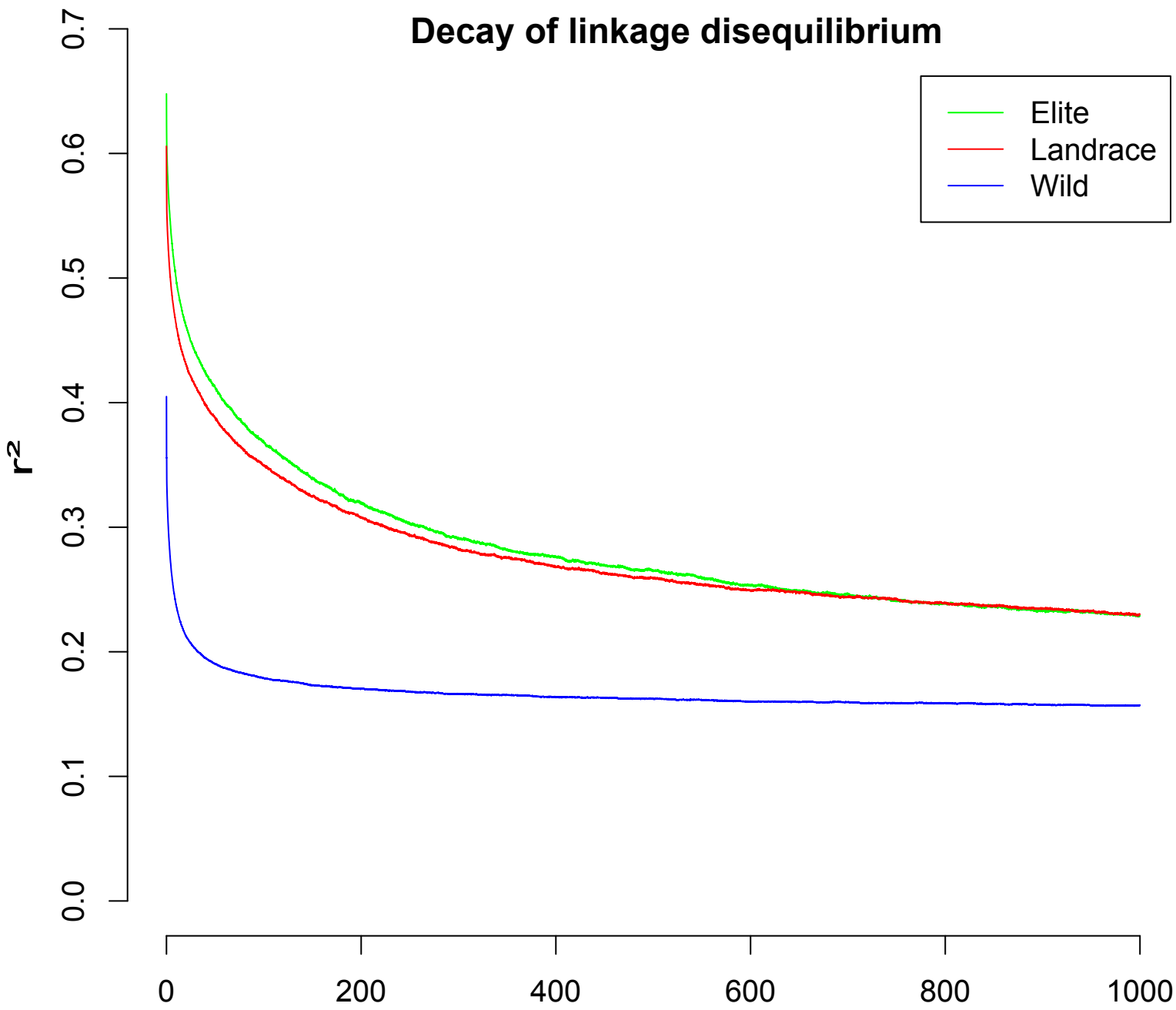

b.

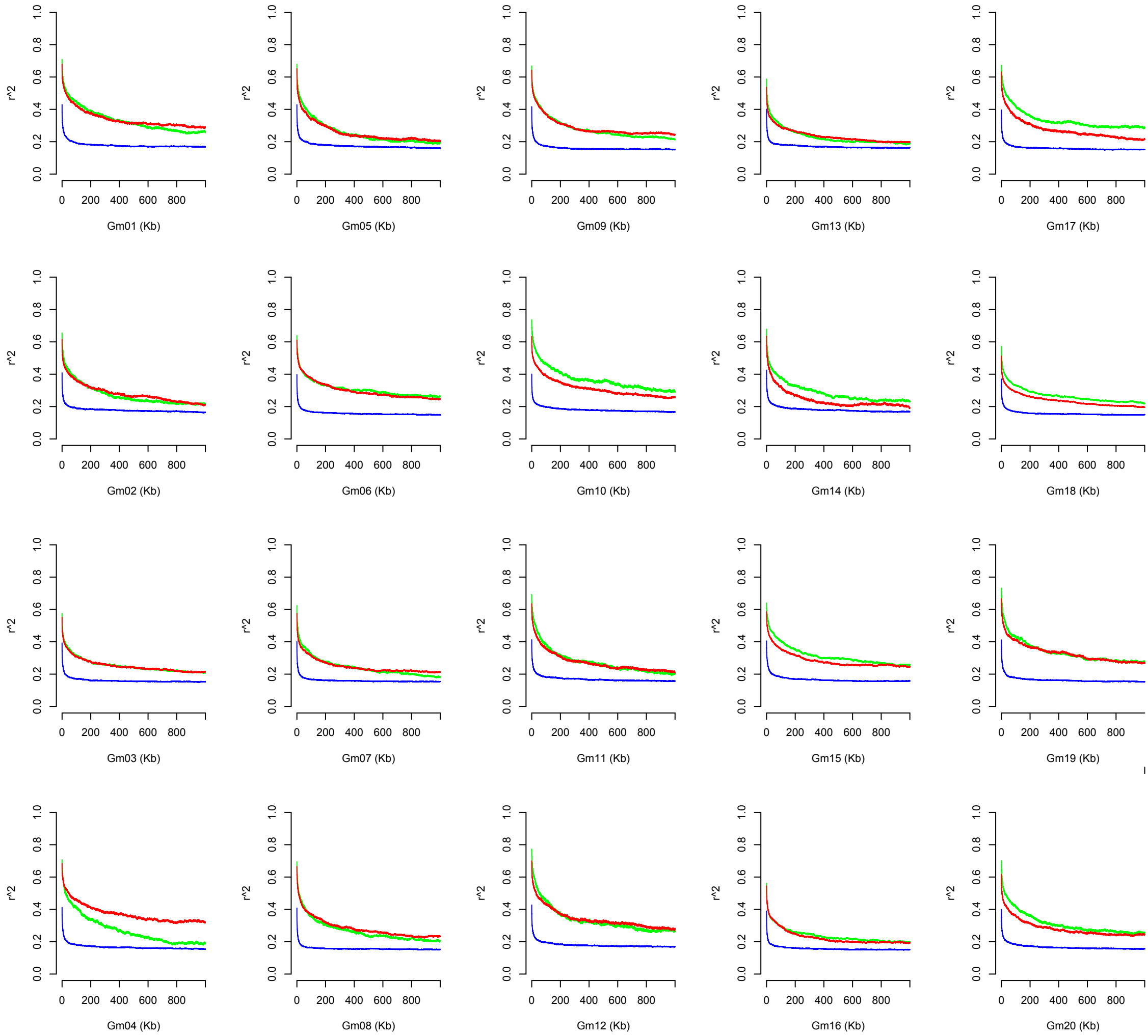

Supplement: Additional file 7 — LD decay determined by squared correlation coefficient of allele frequencies (r2) in against distance among three soybean gene pools on whole genome level (a) and at each chromosome (b). [file 1471-2164-14-579-S7.pdf]

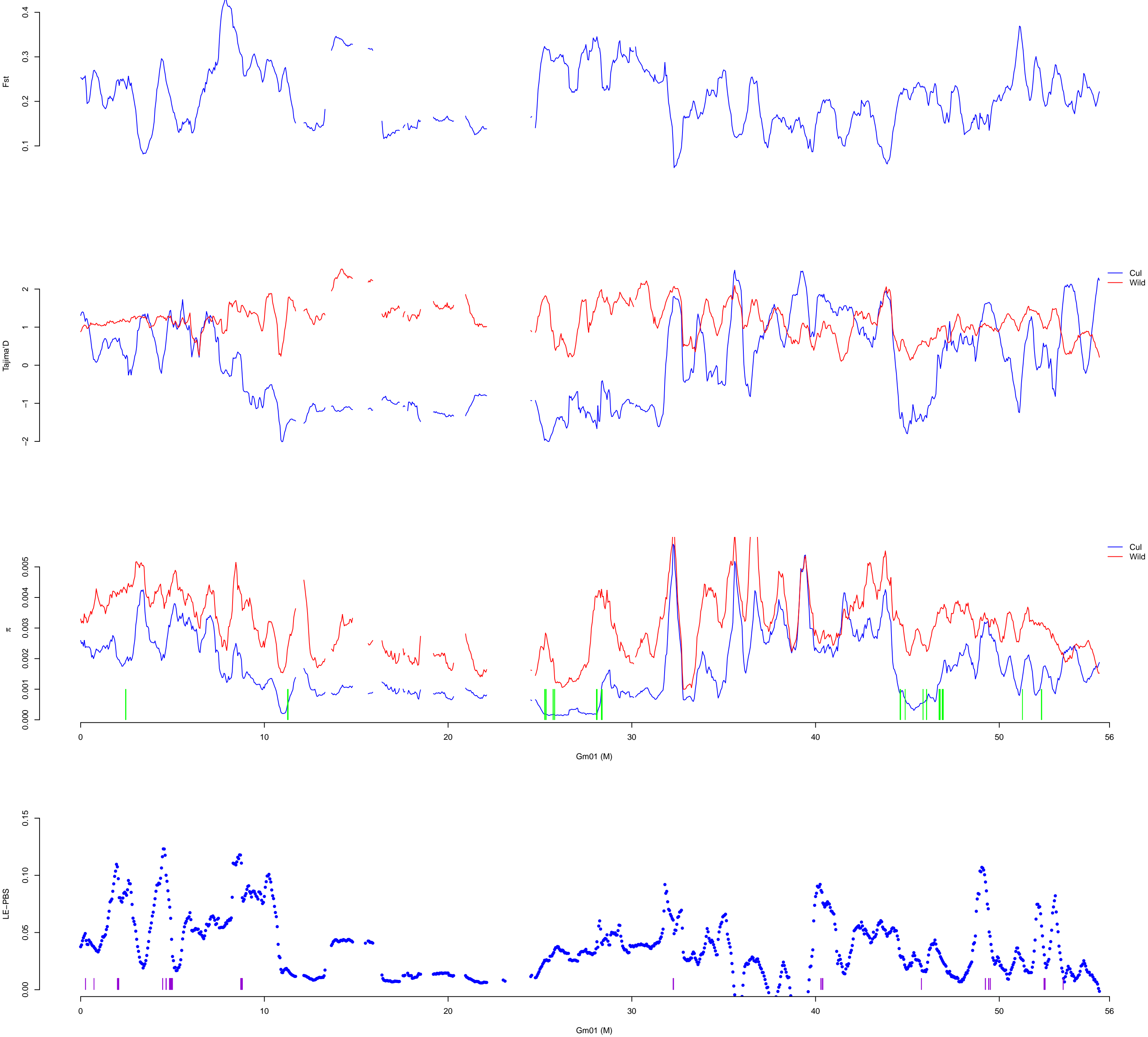

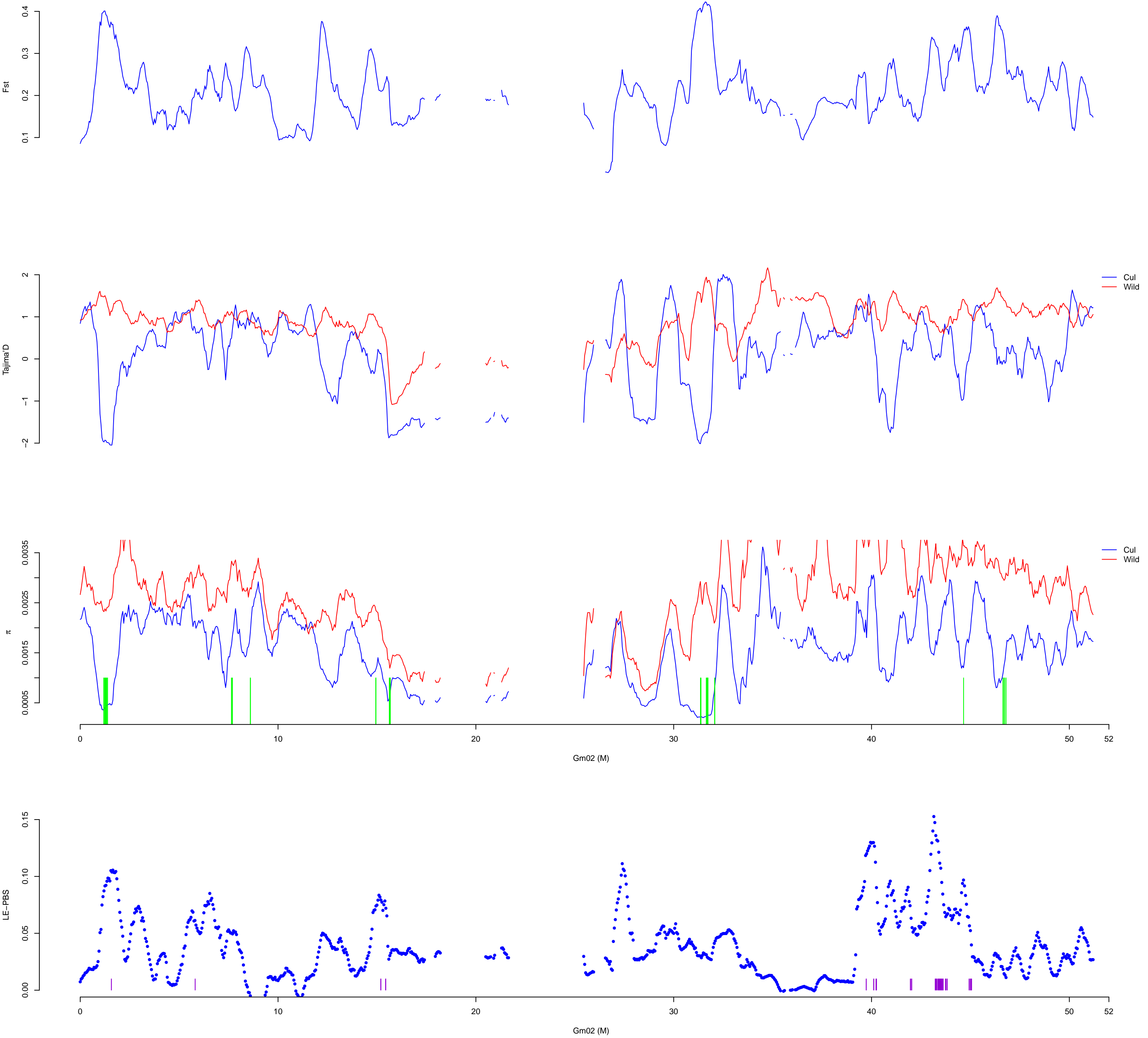

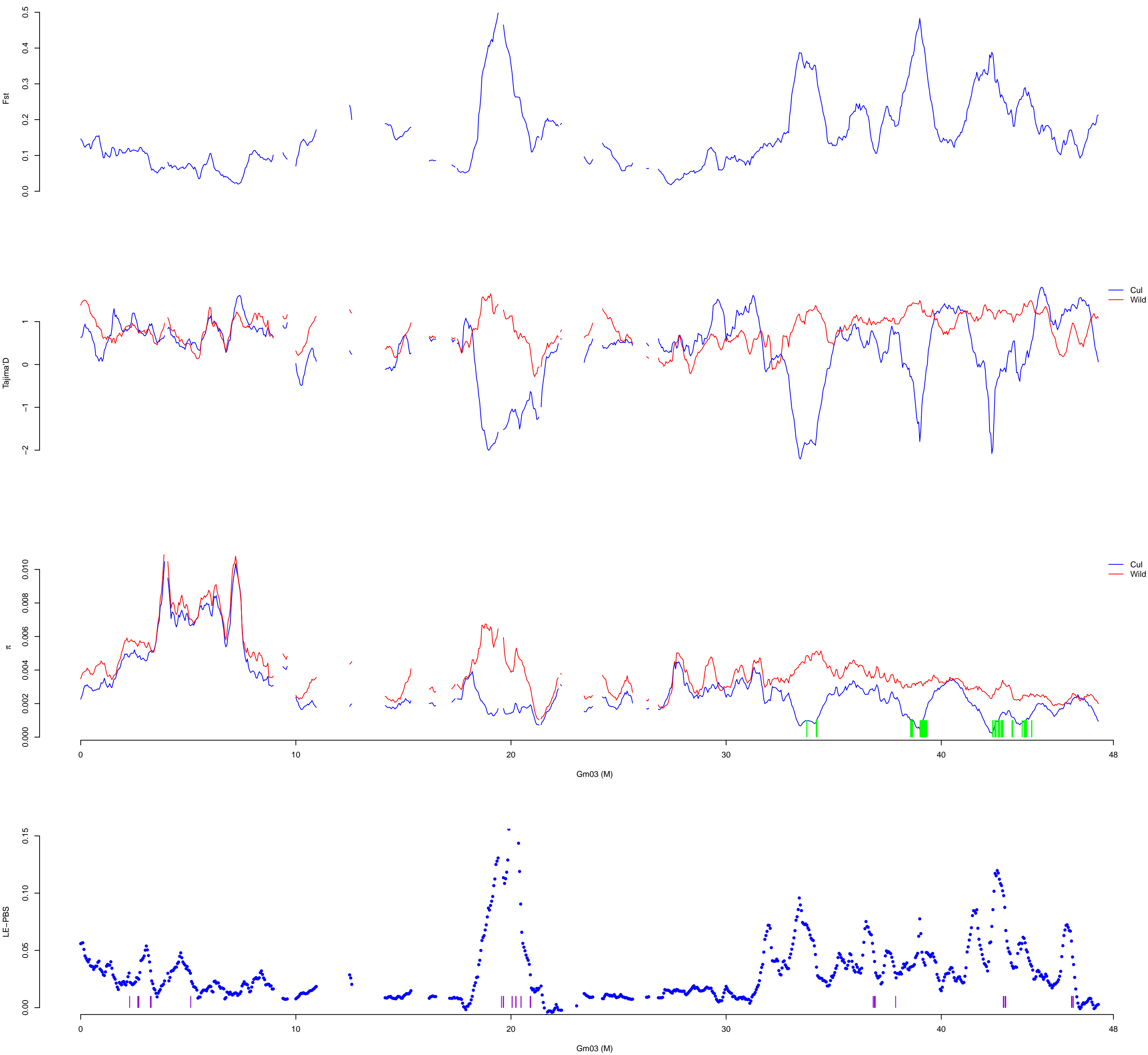

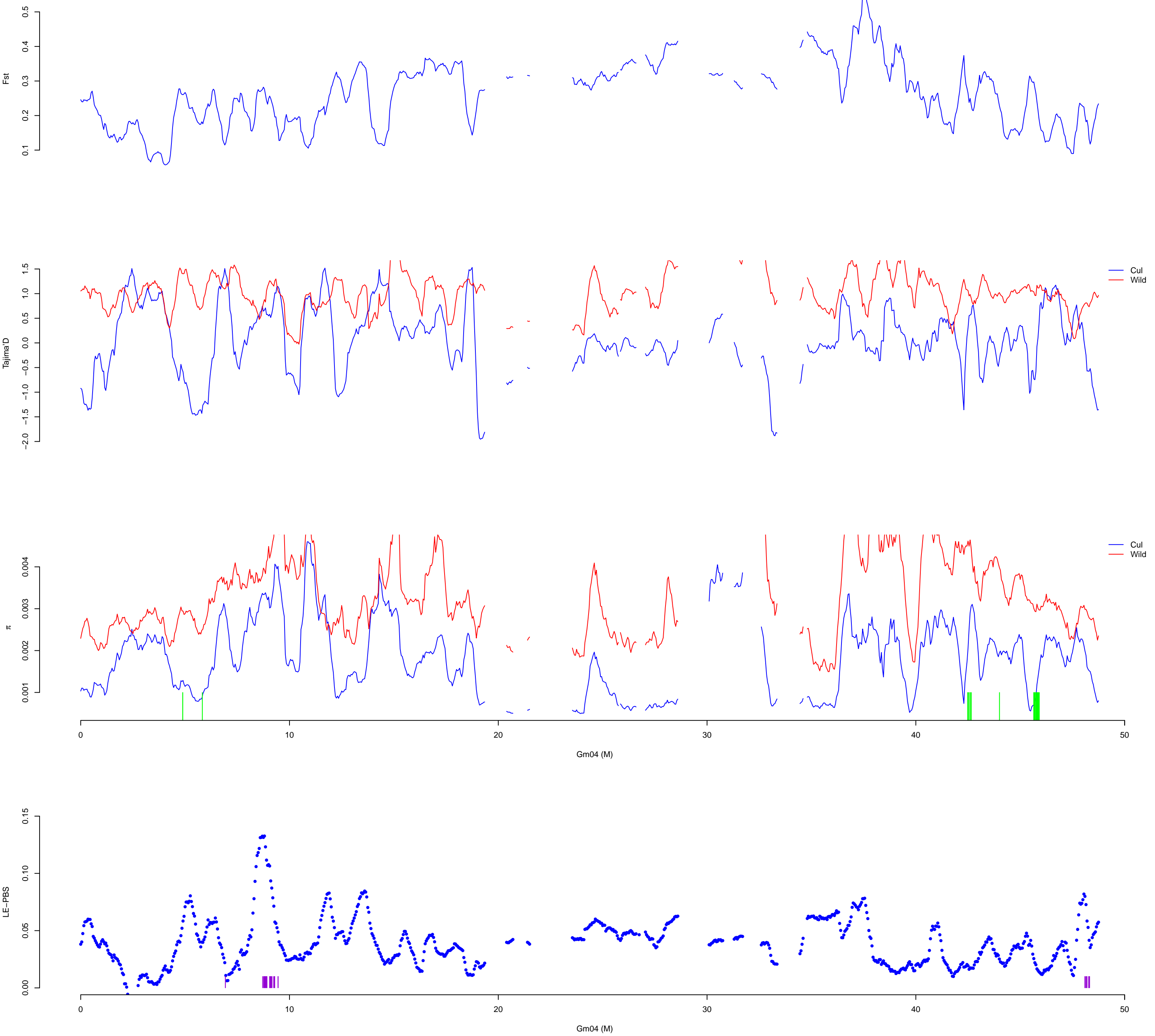

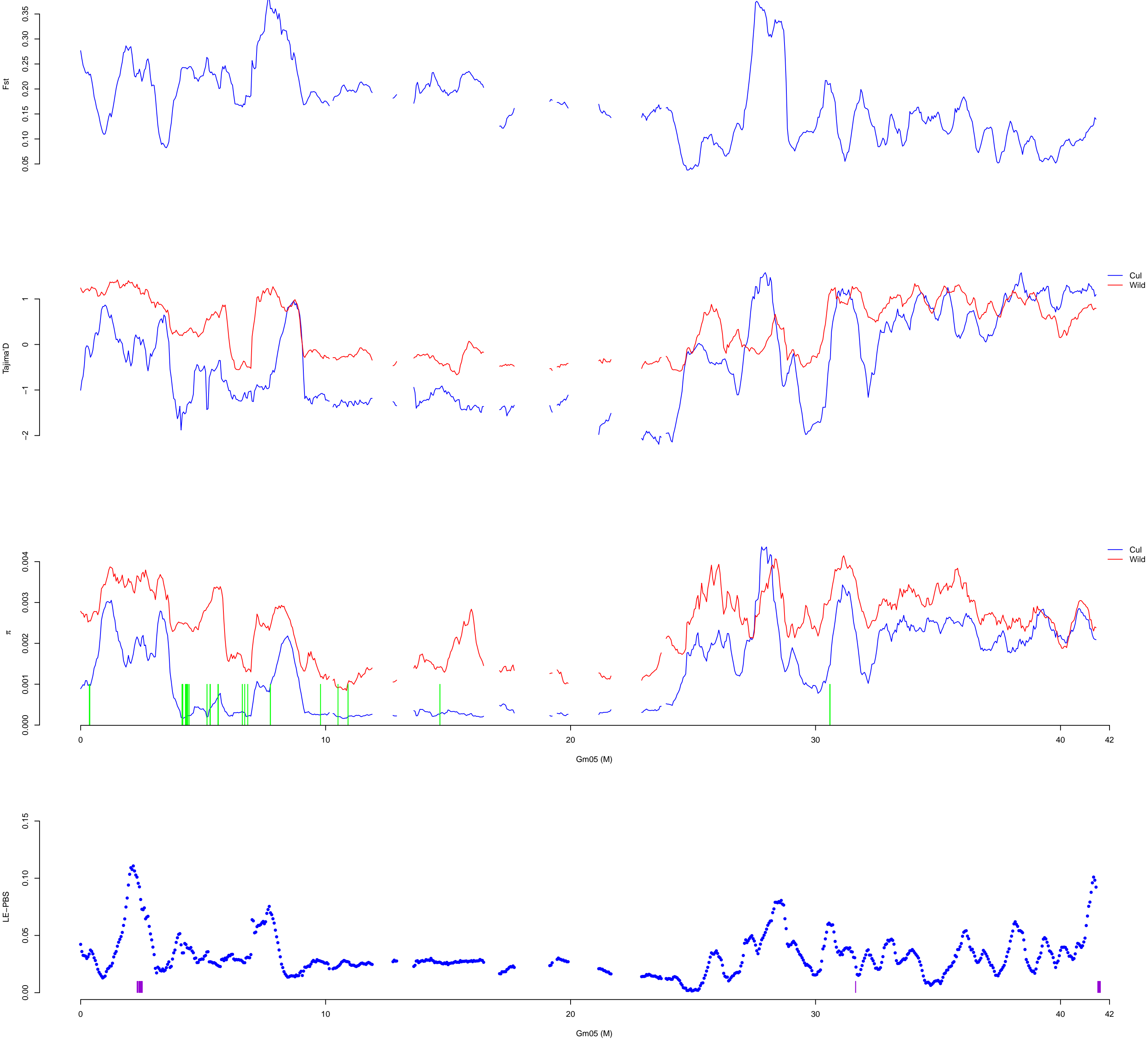

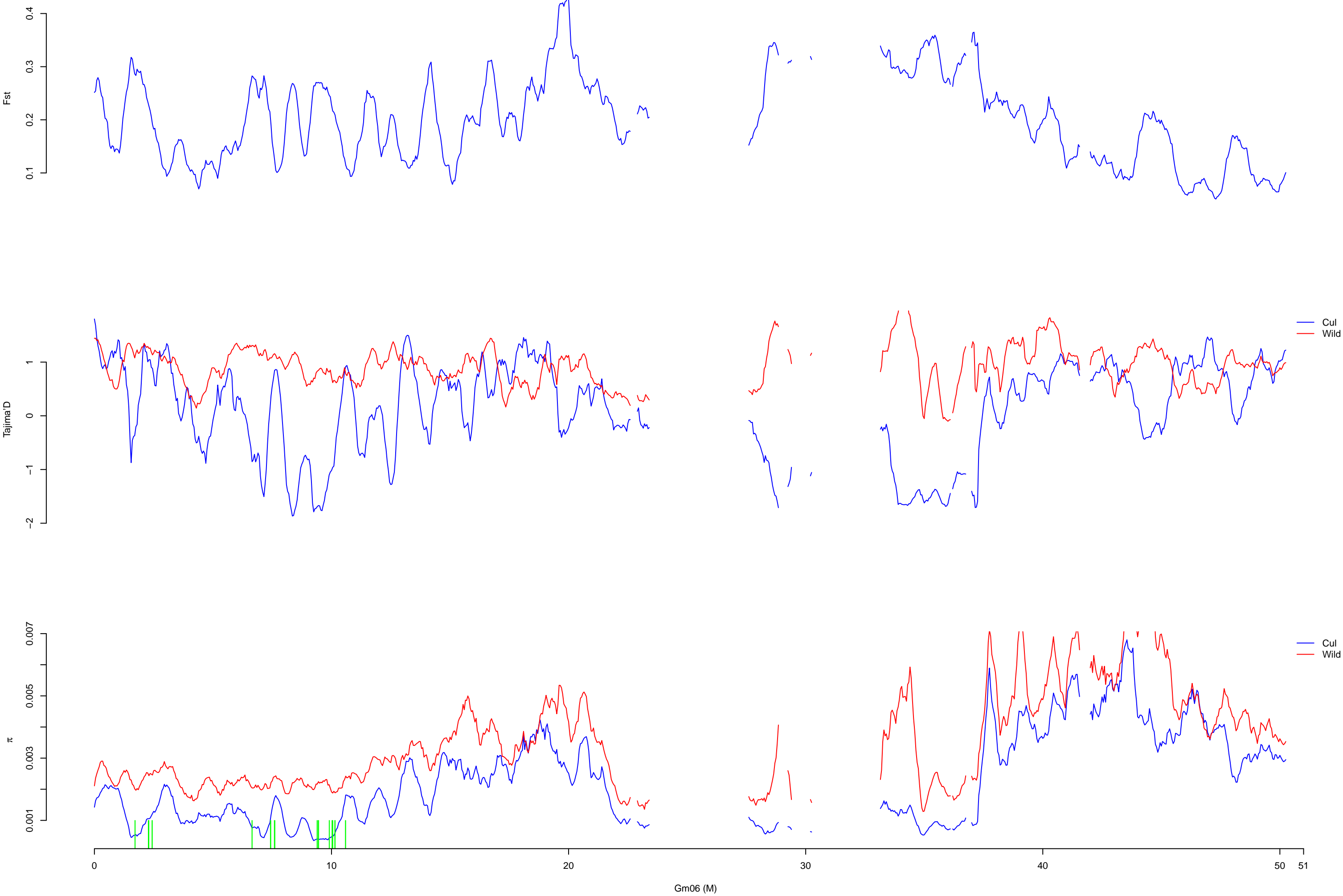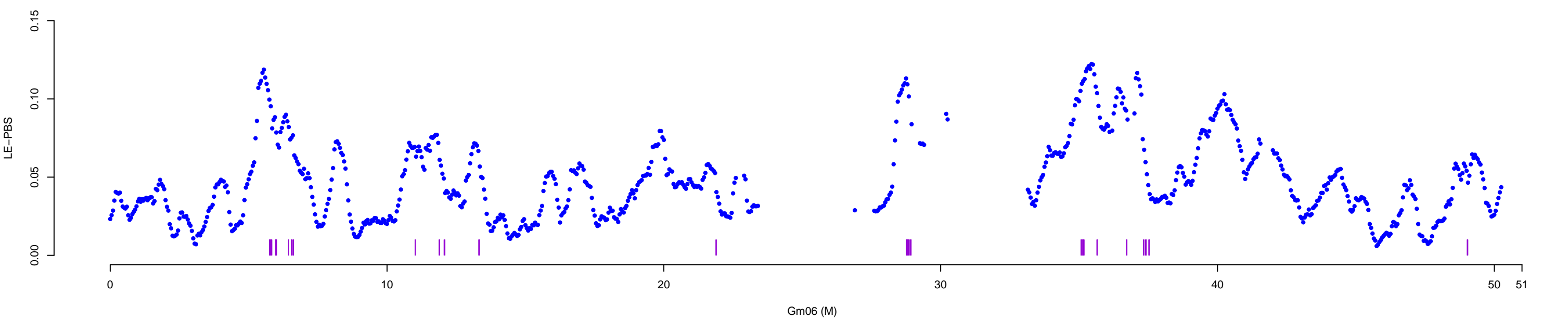

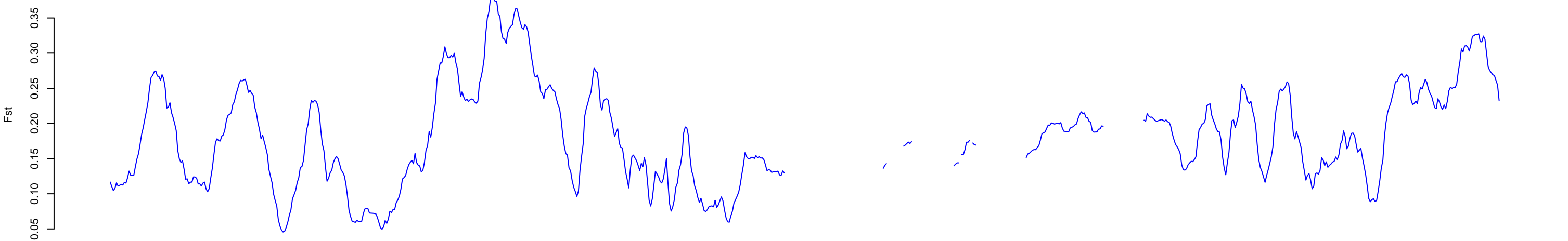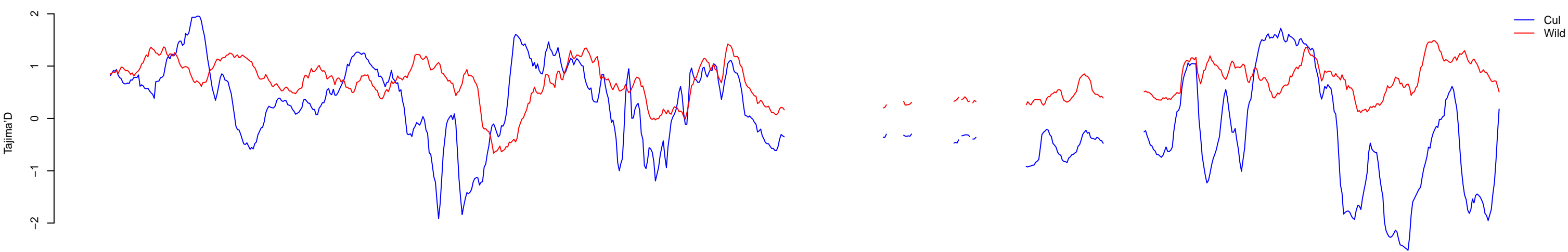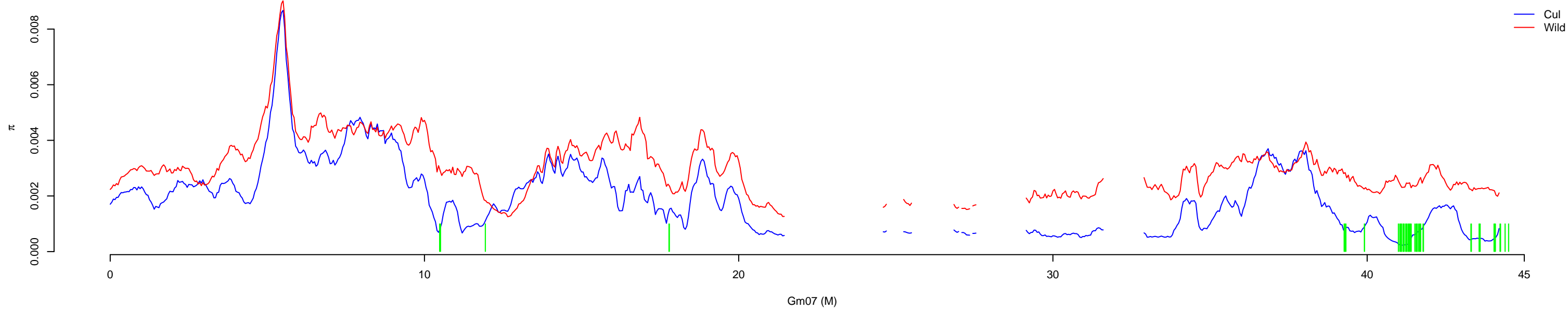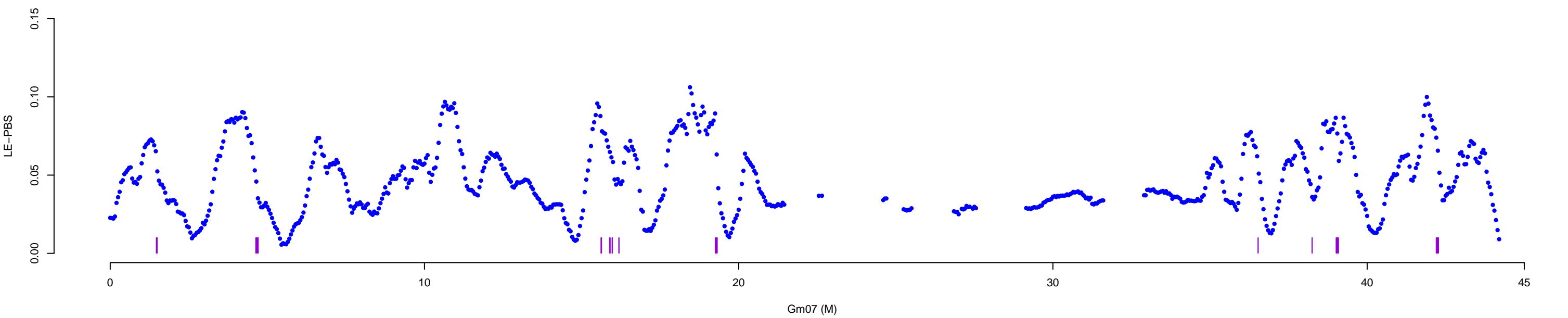

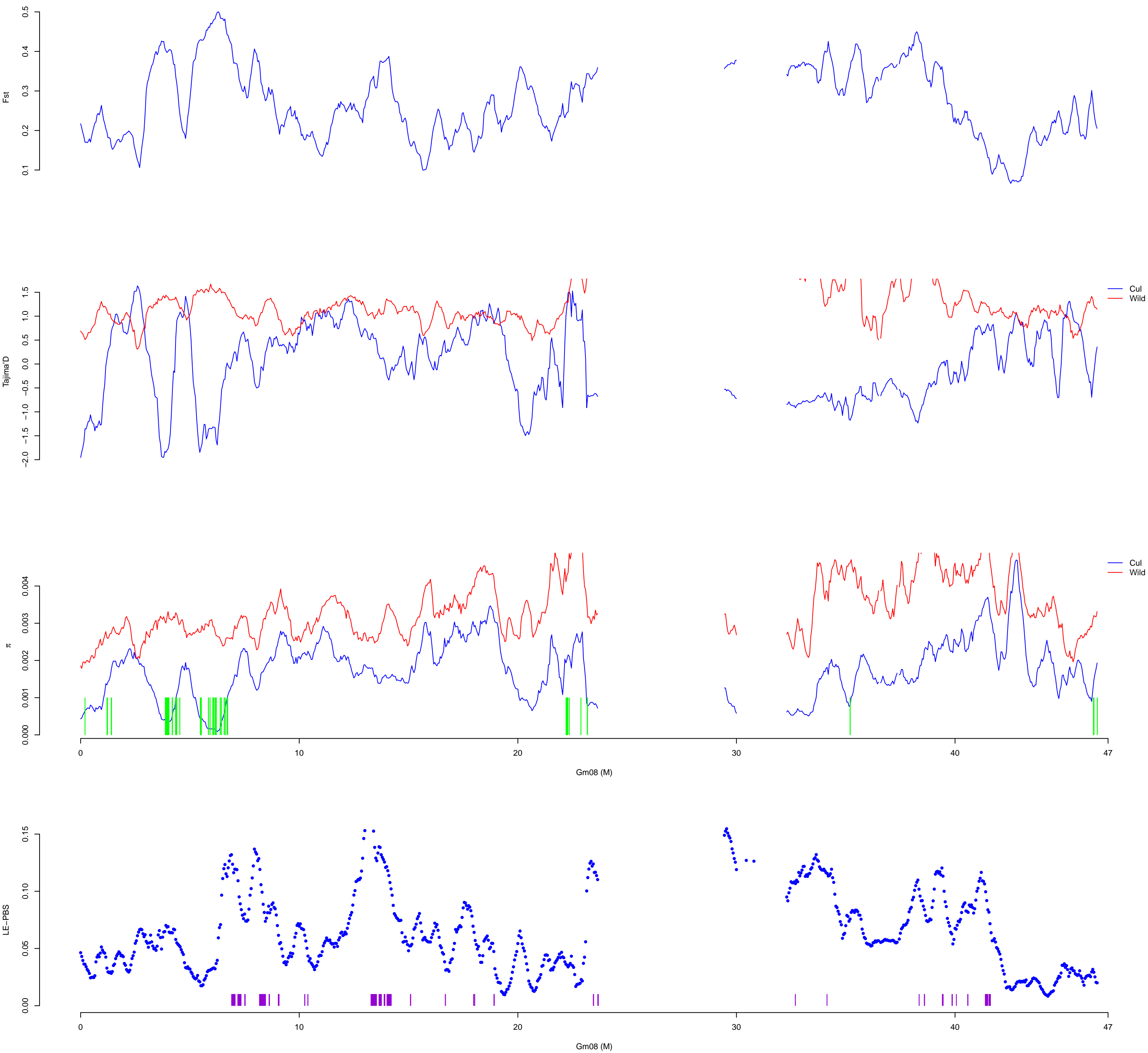

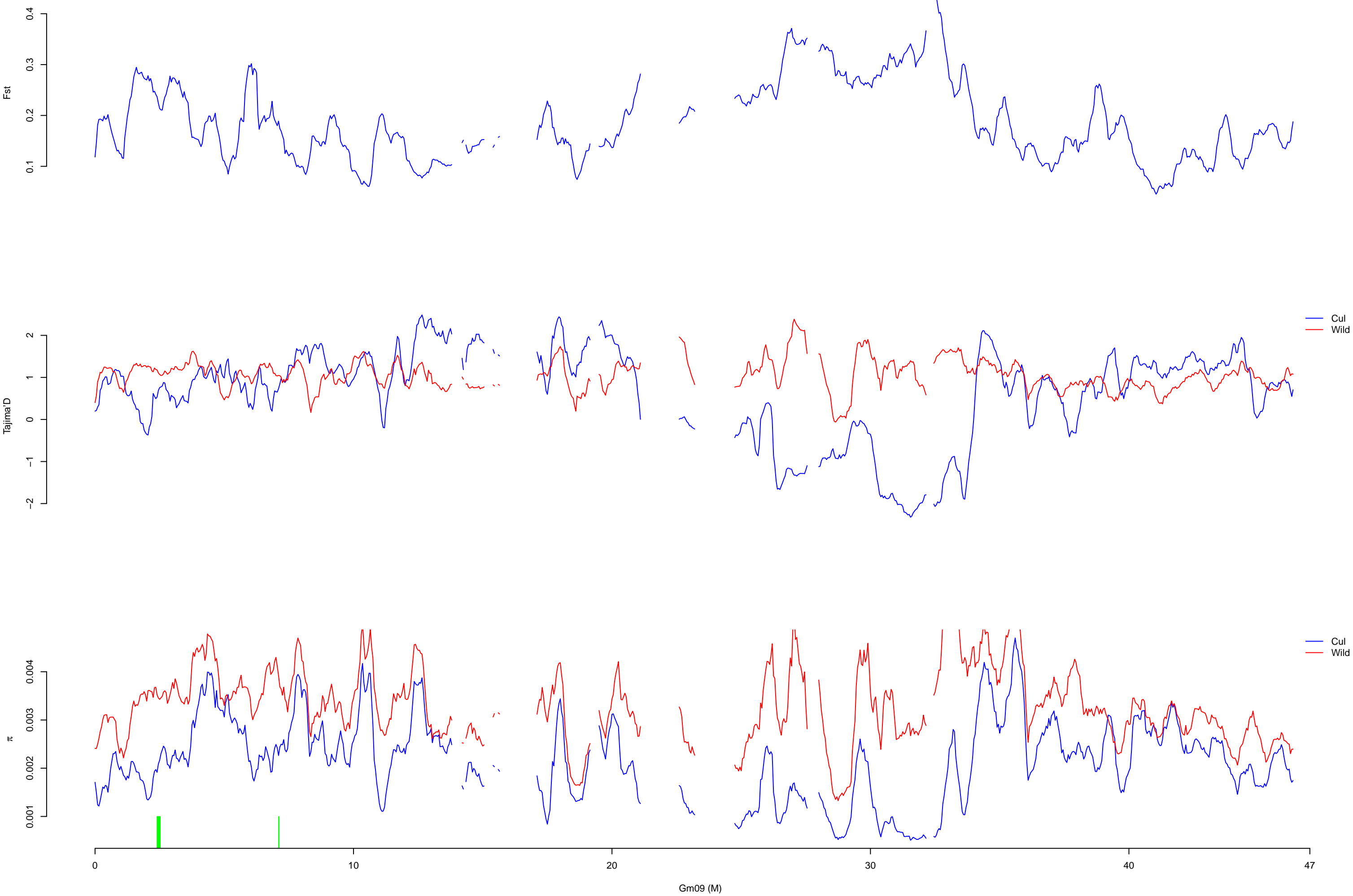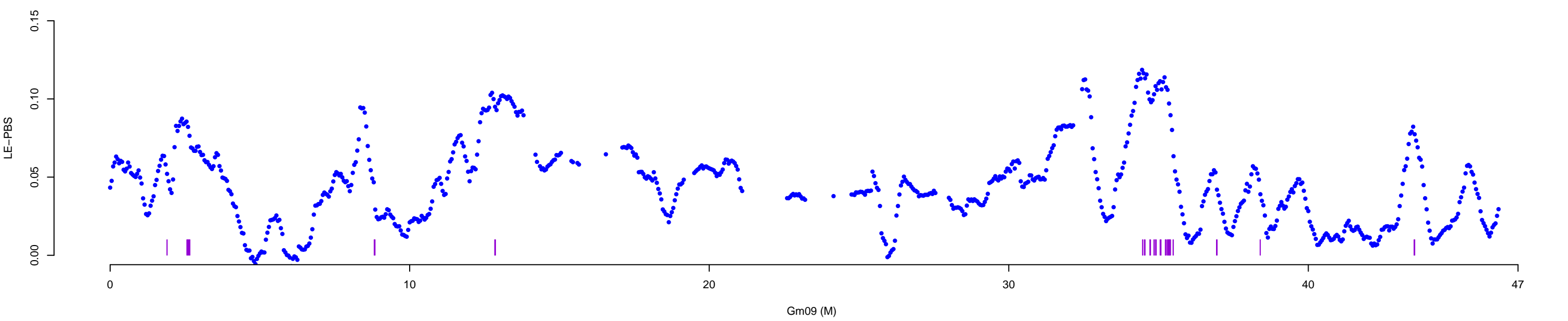

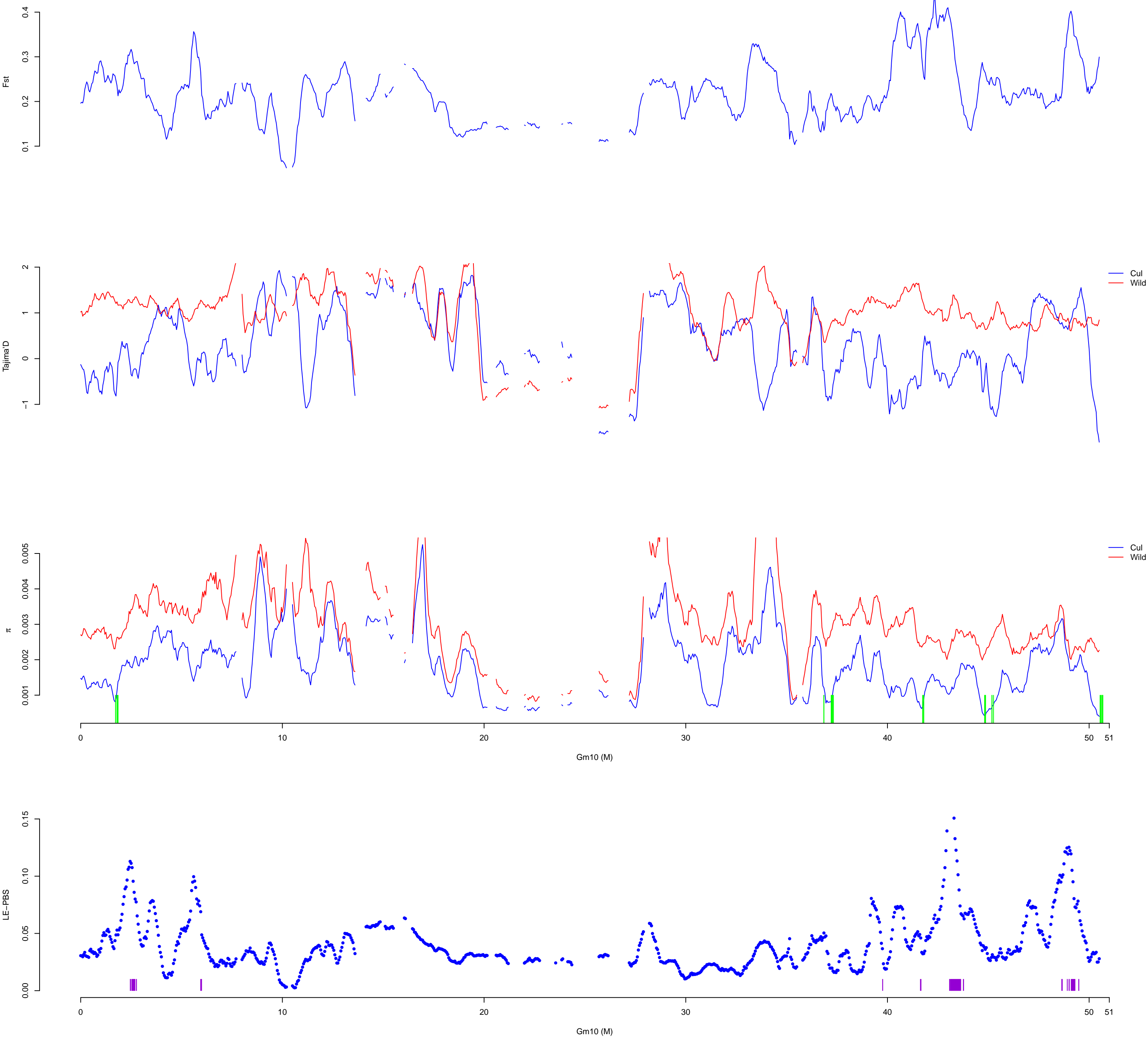

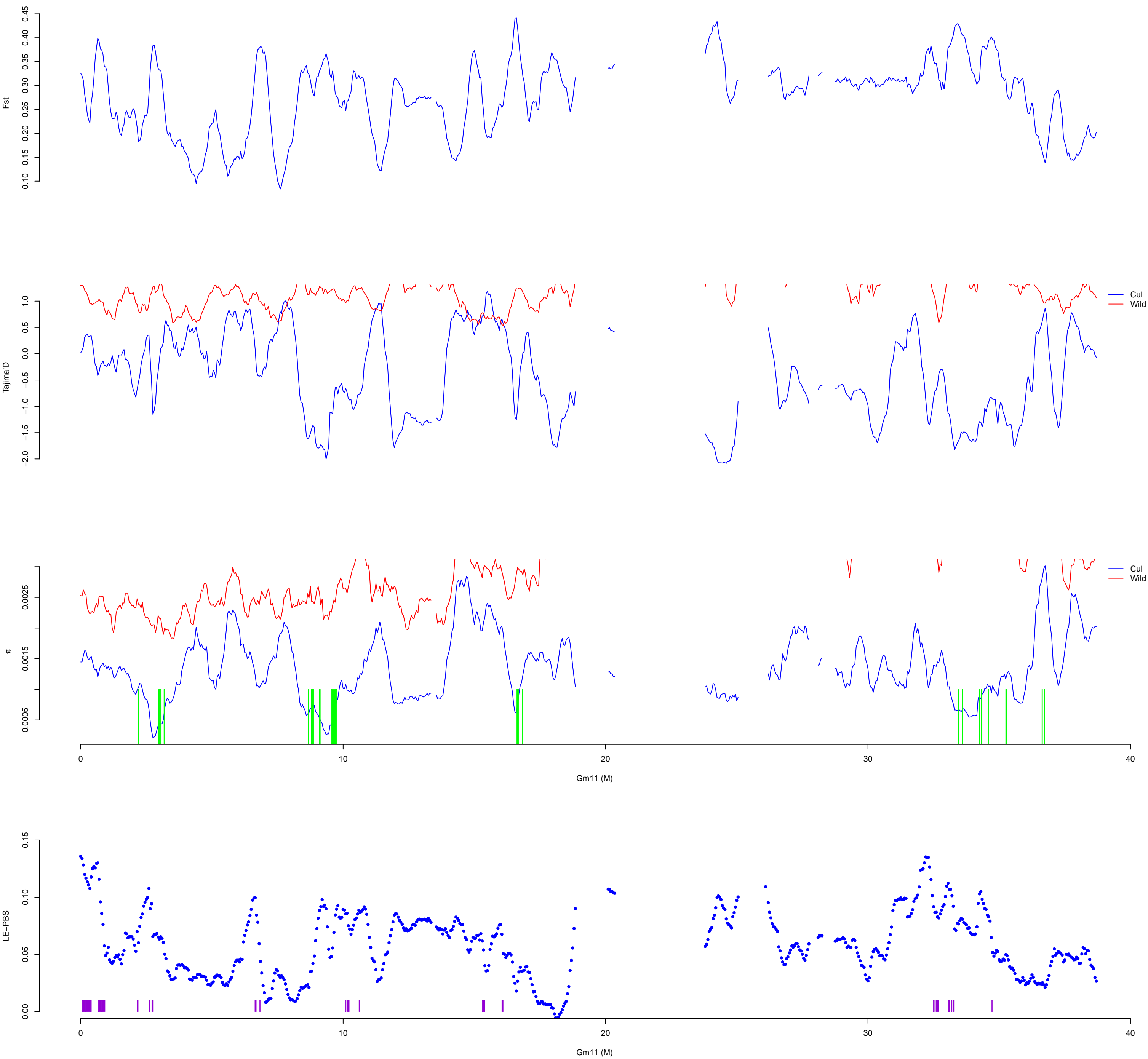

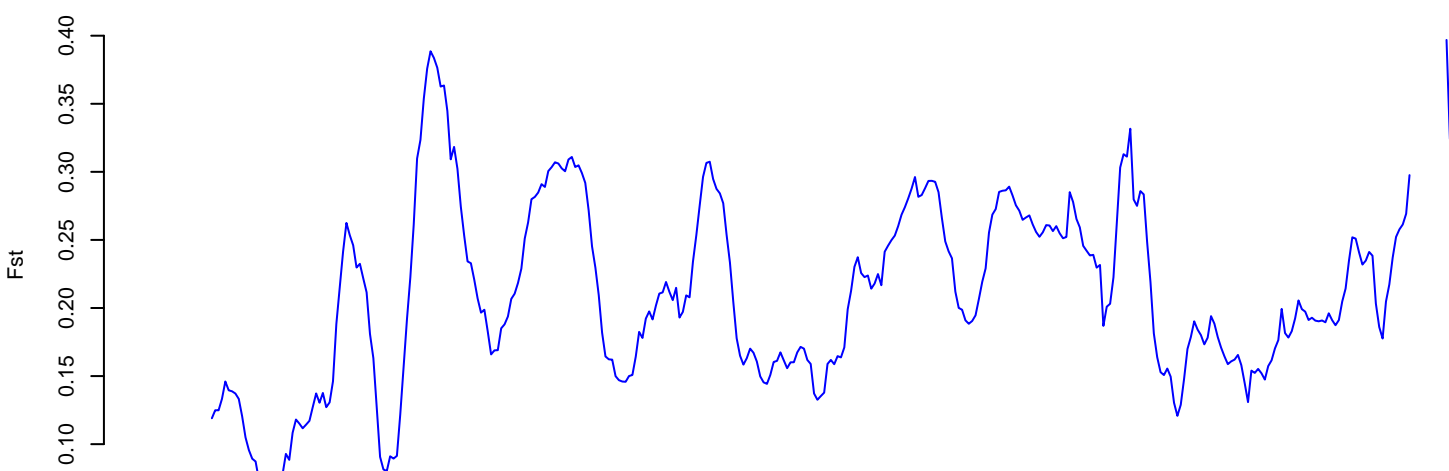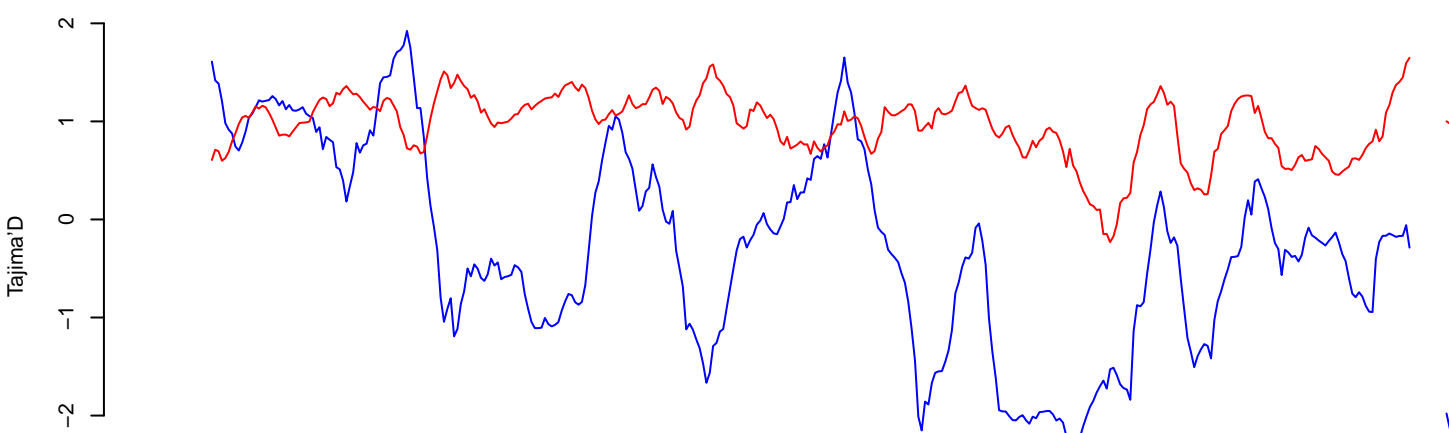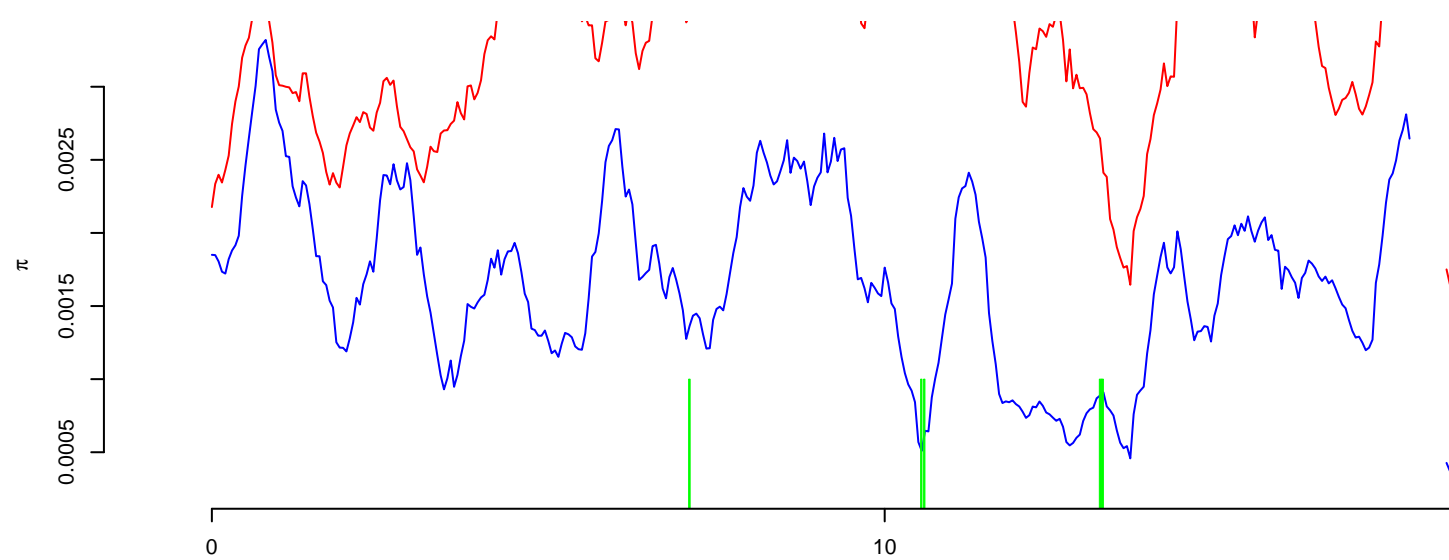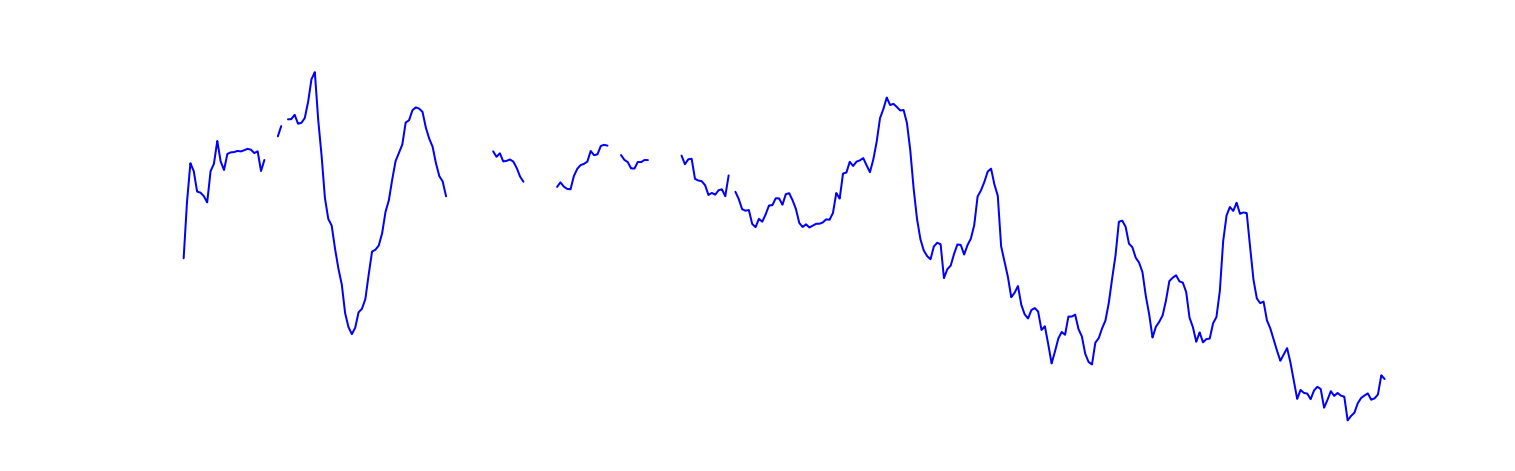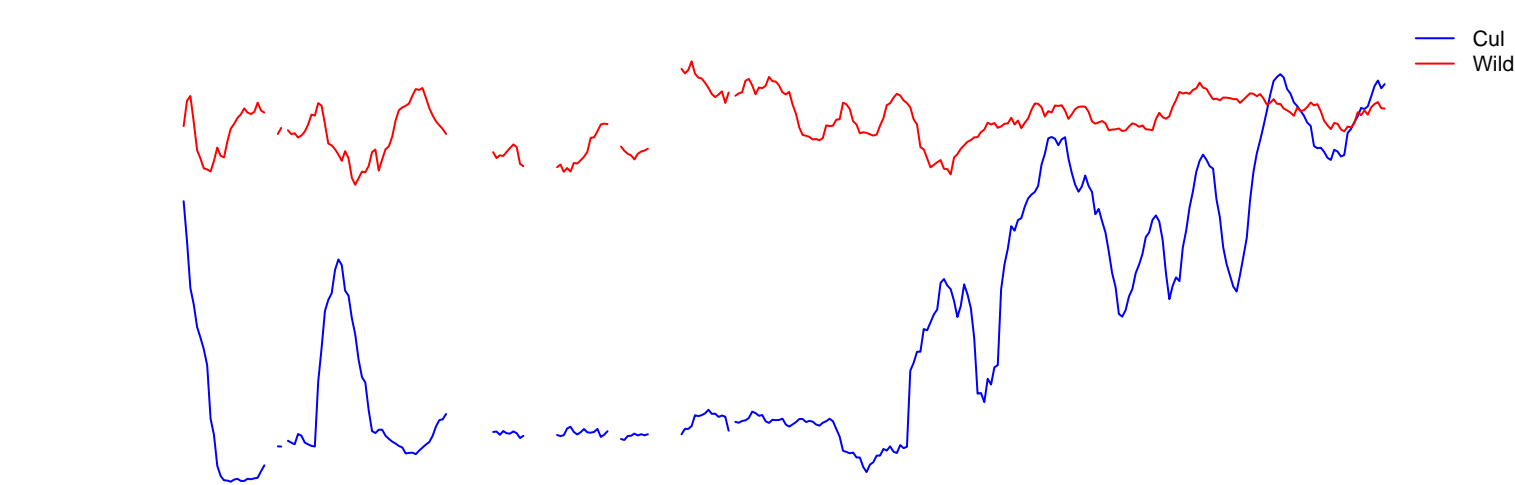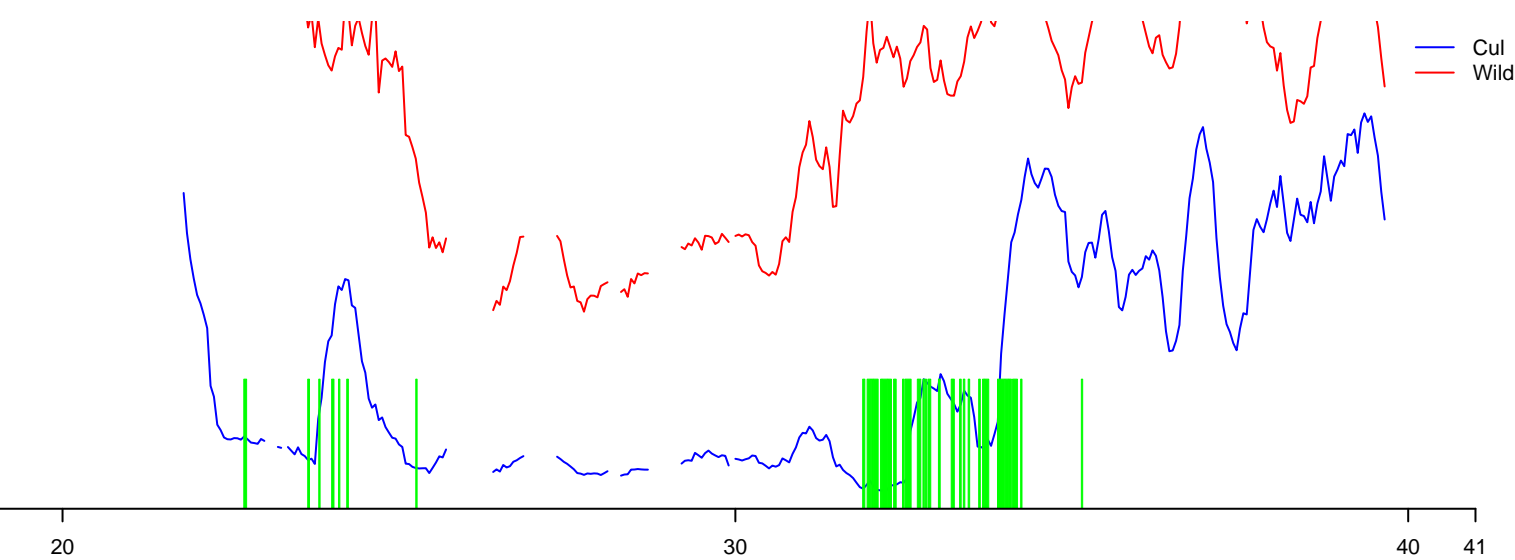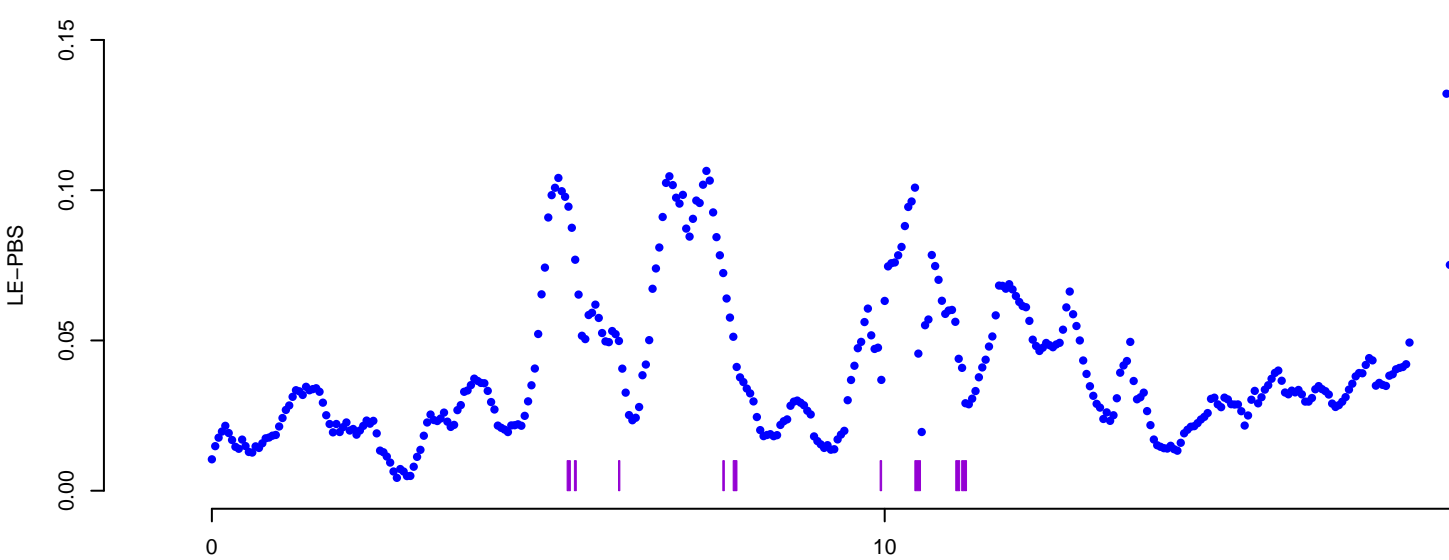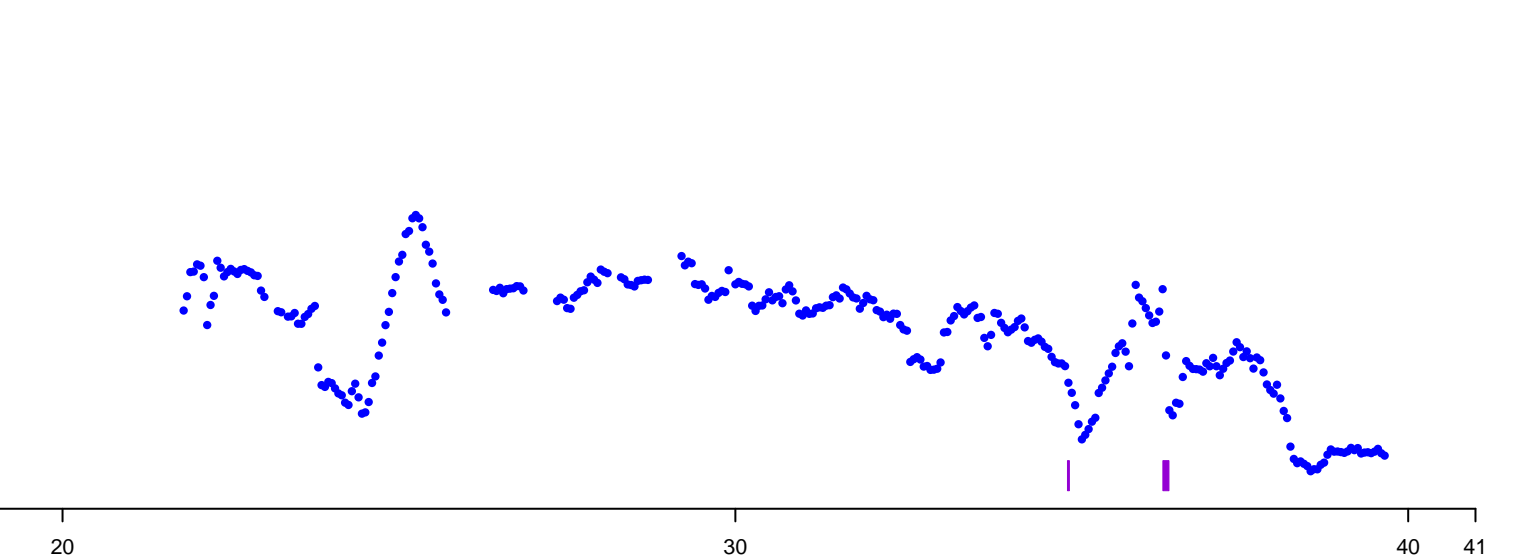

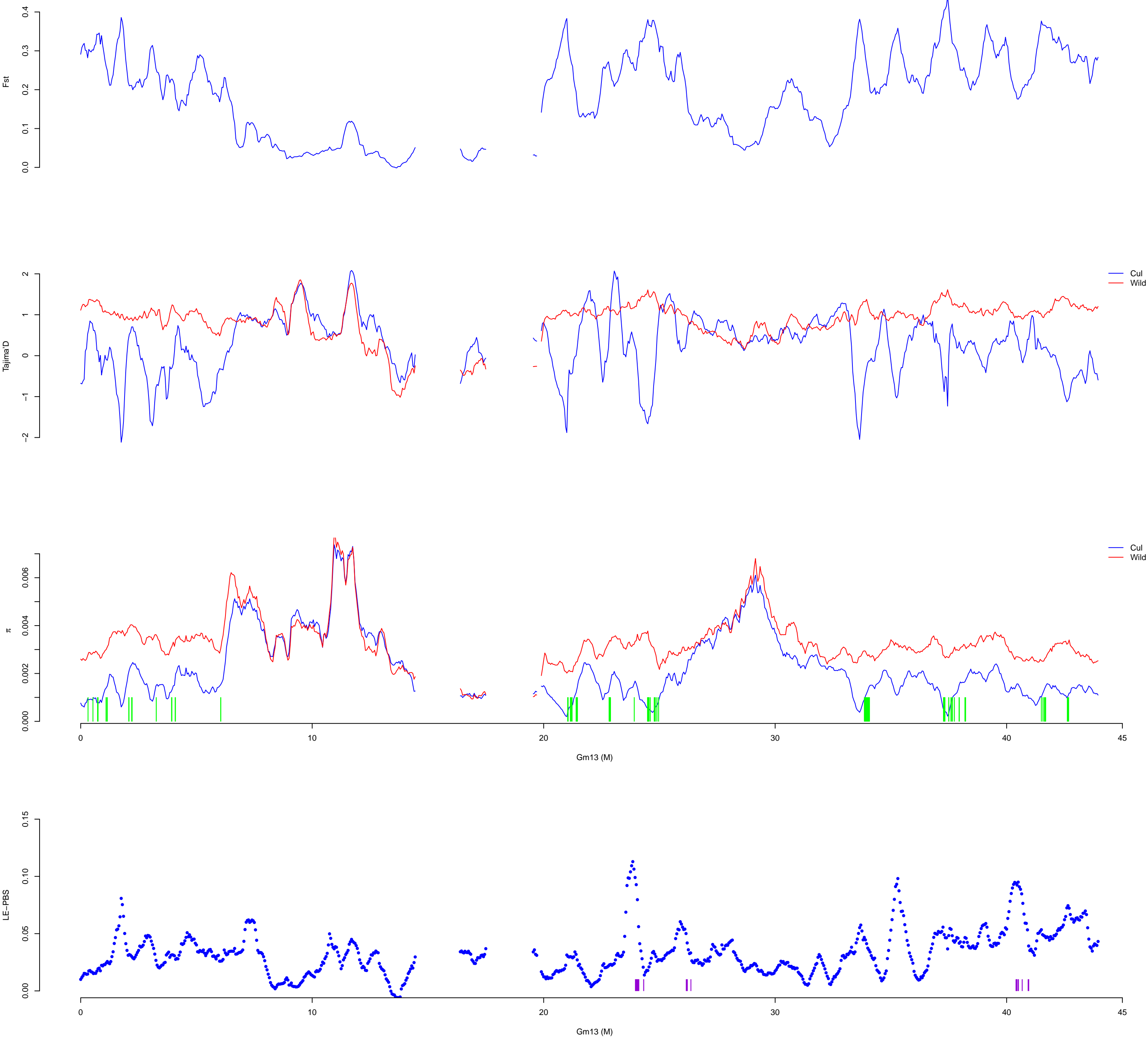

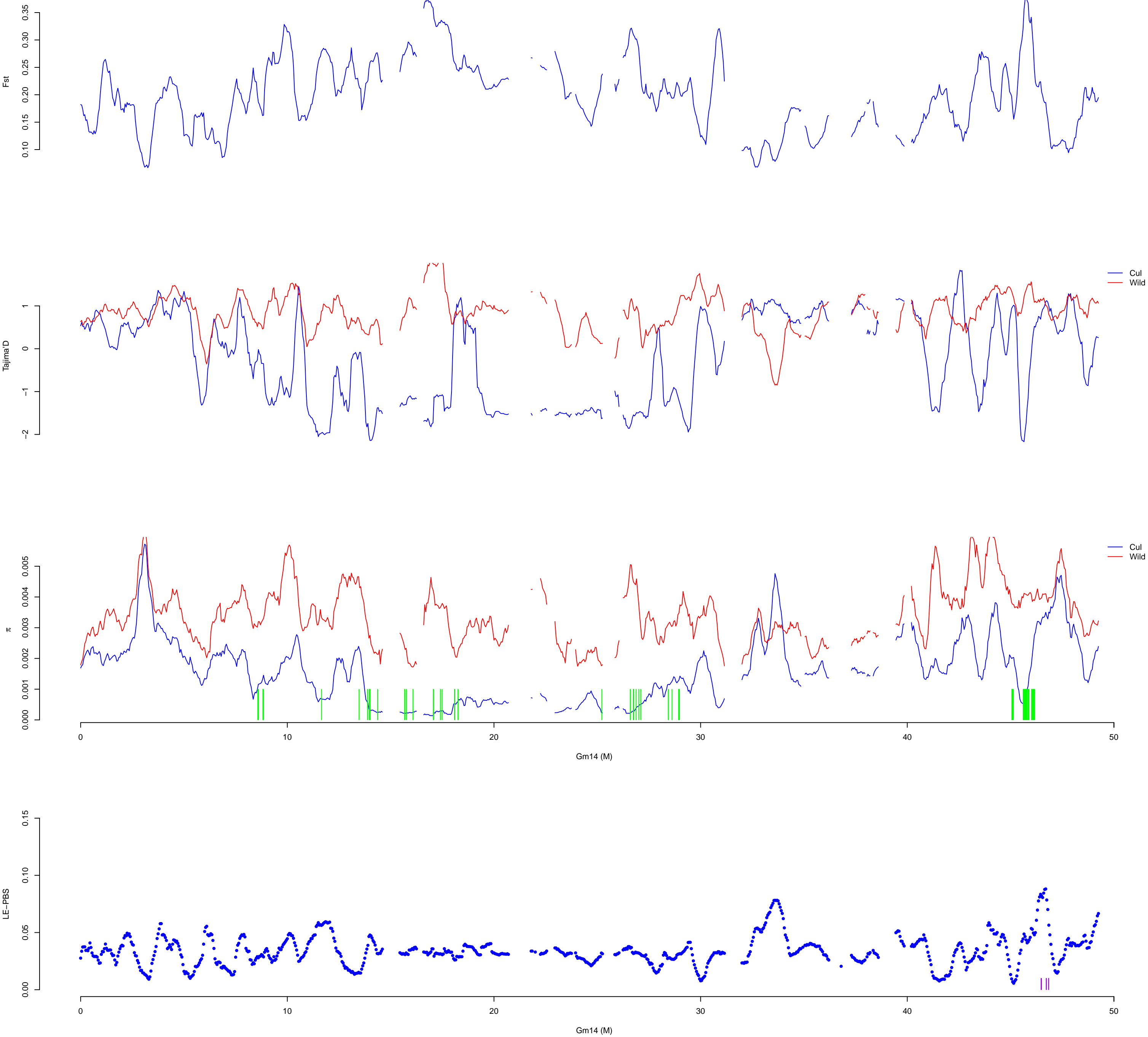

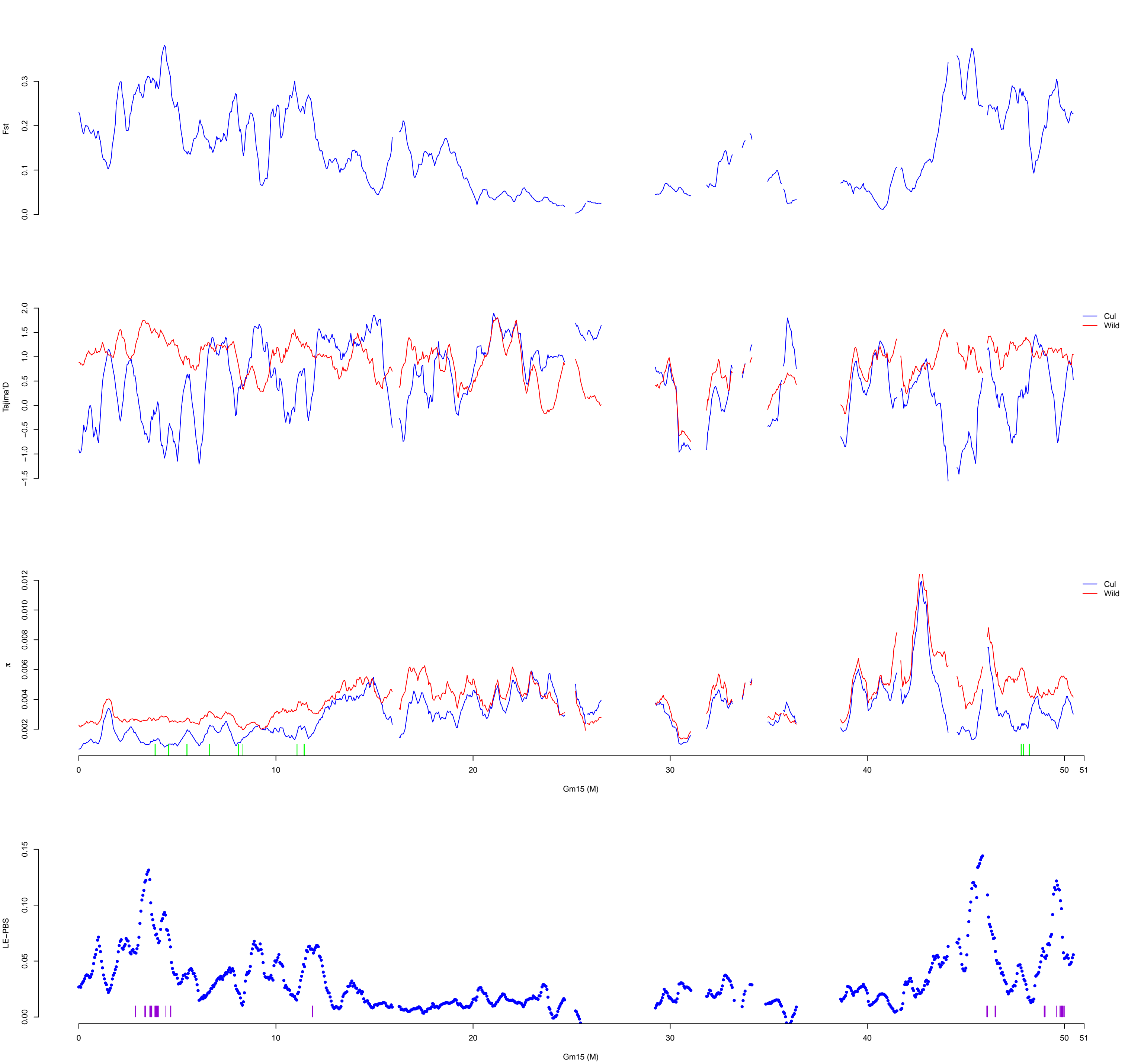

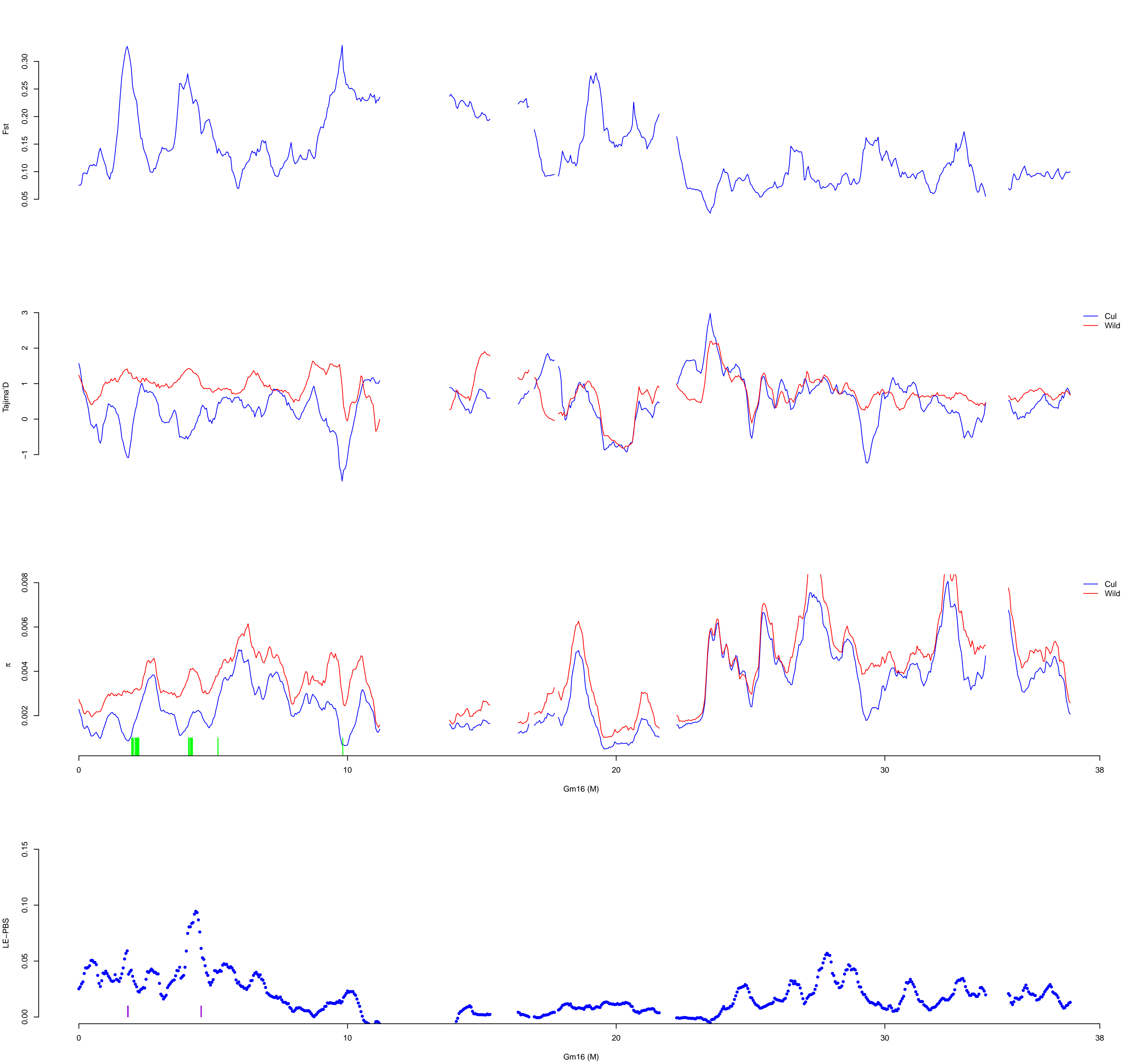

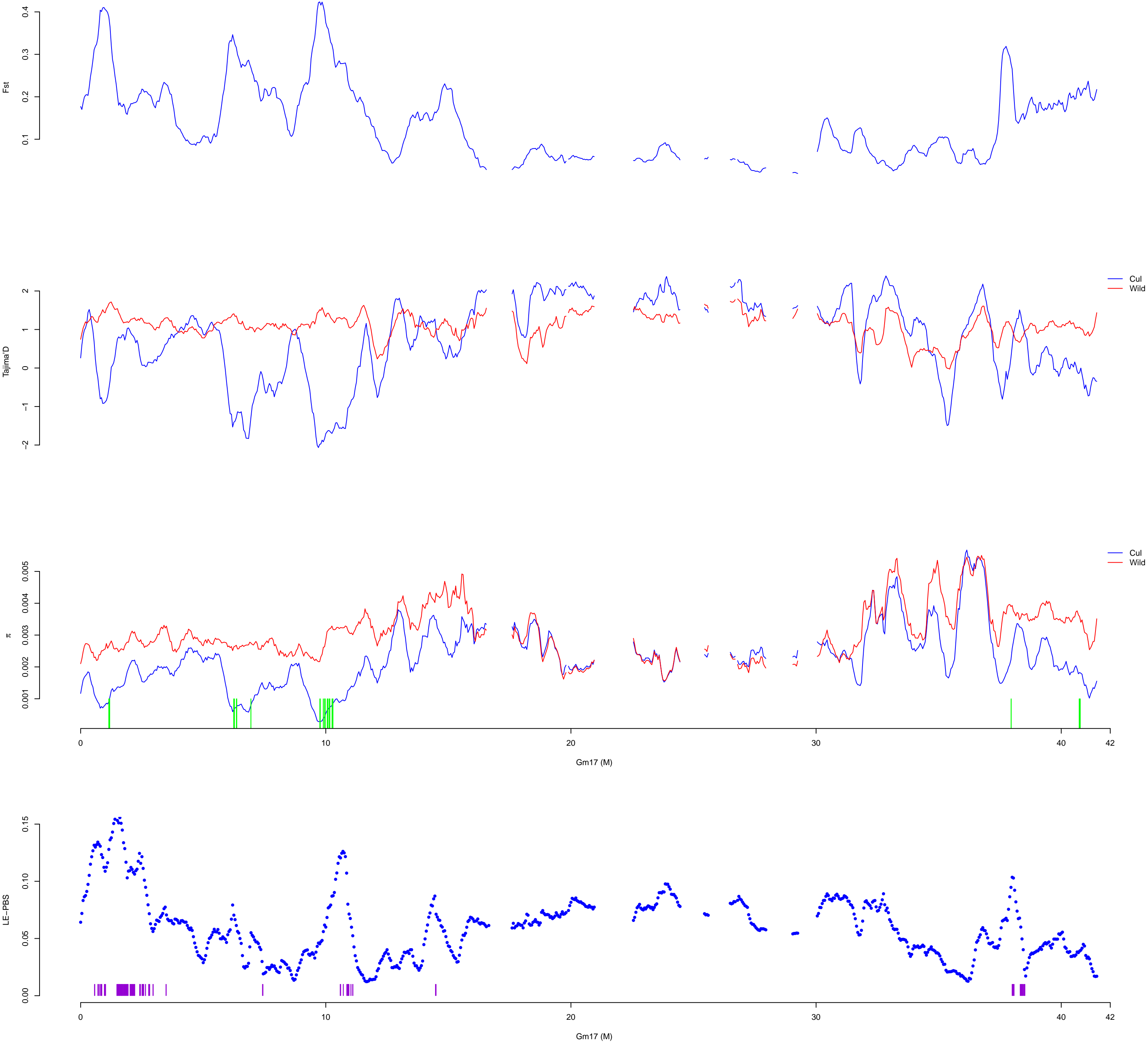

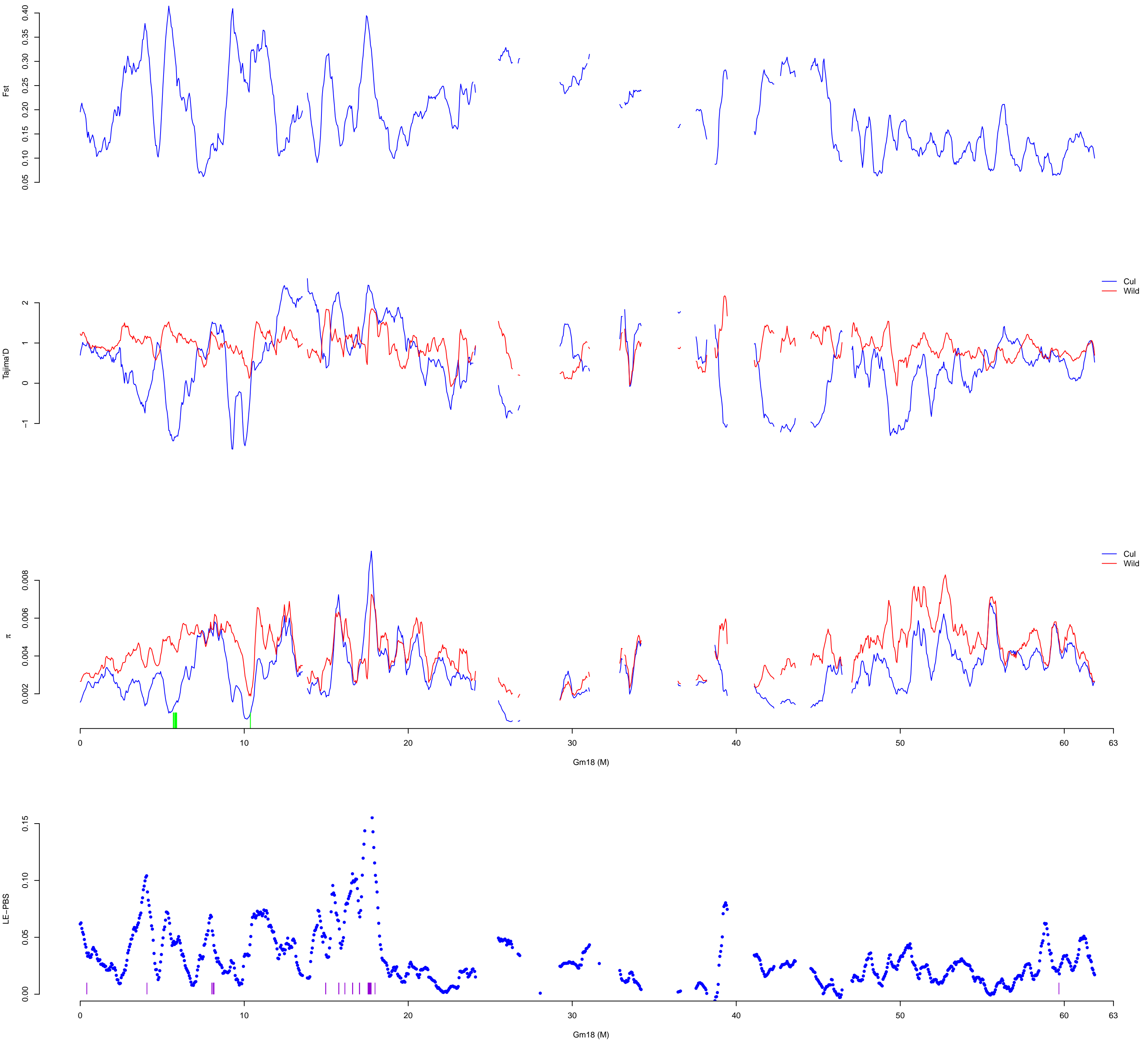

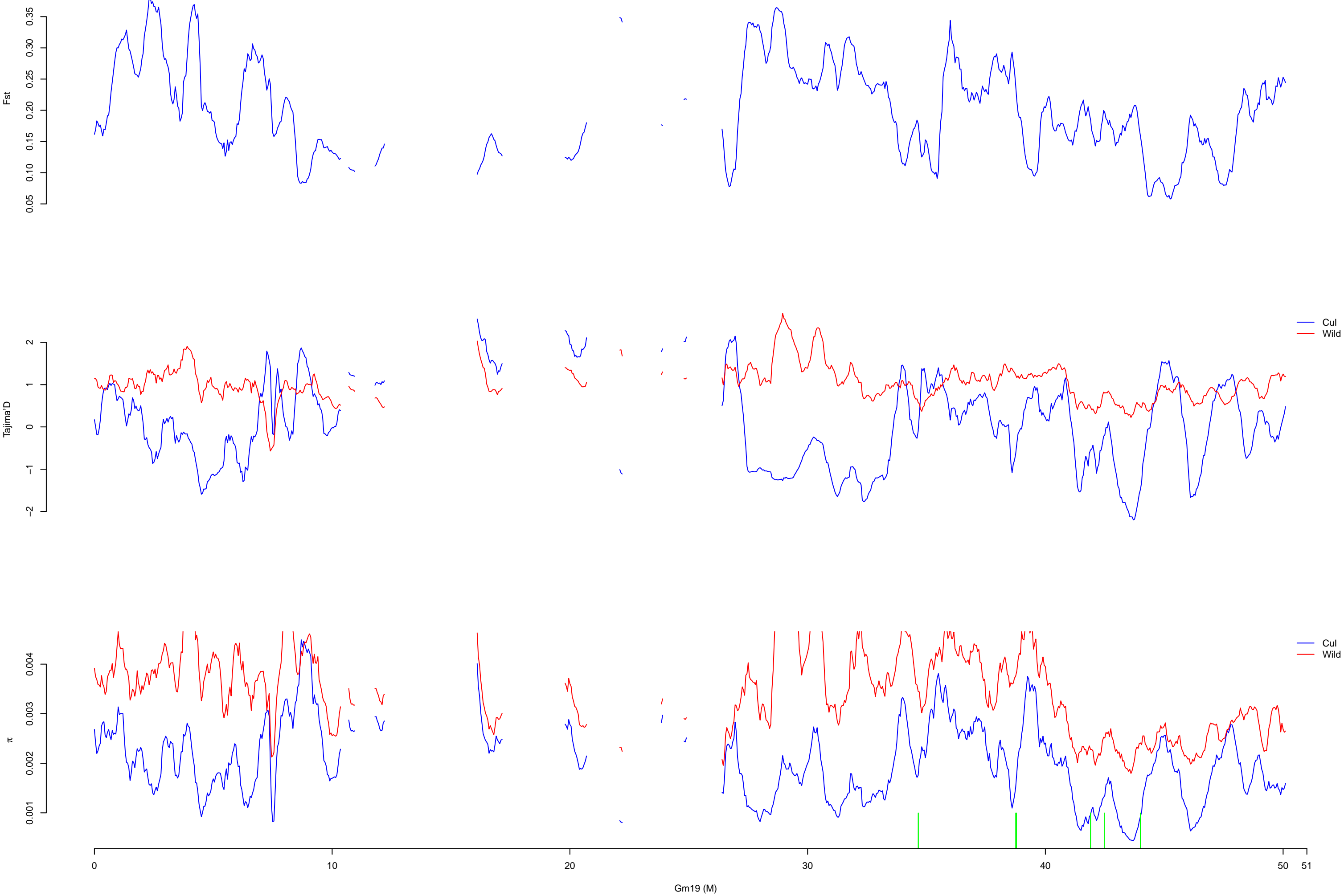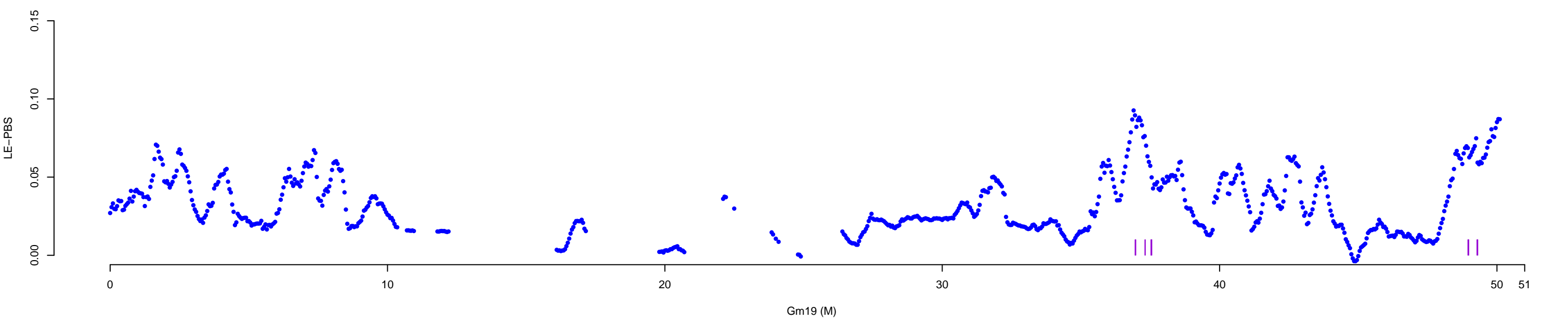

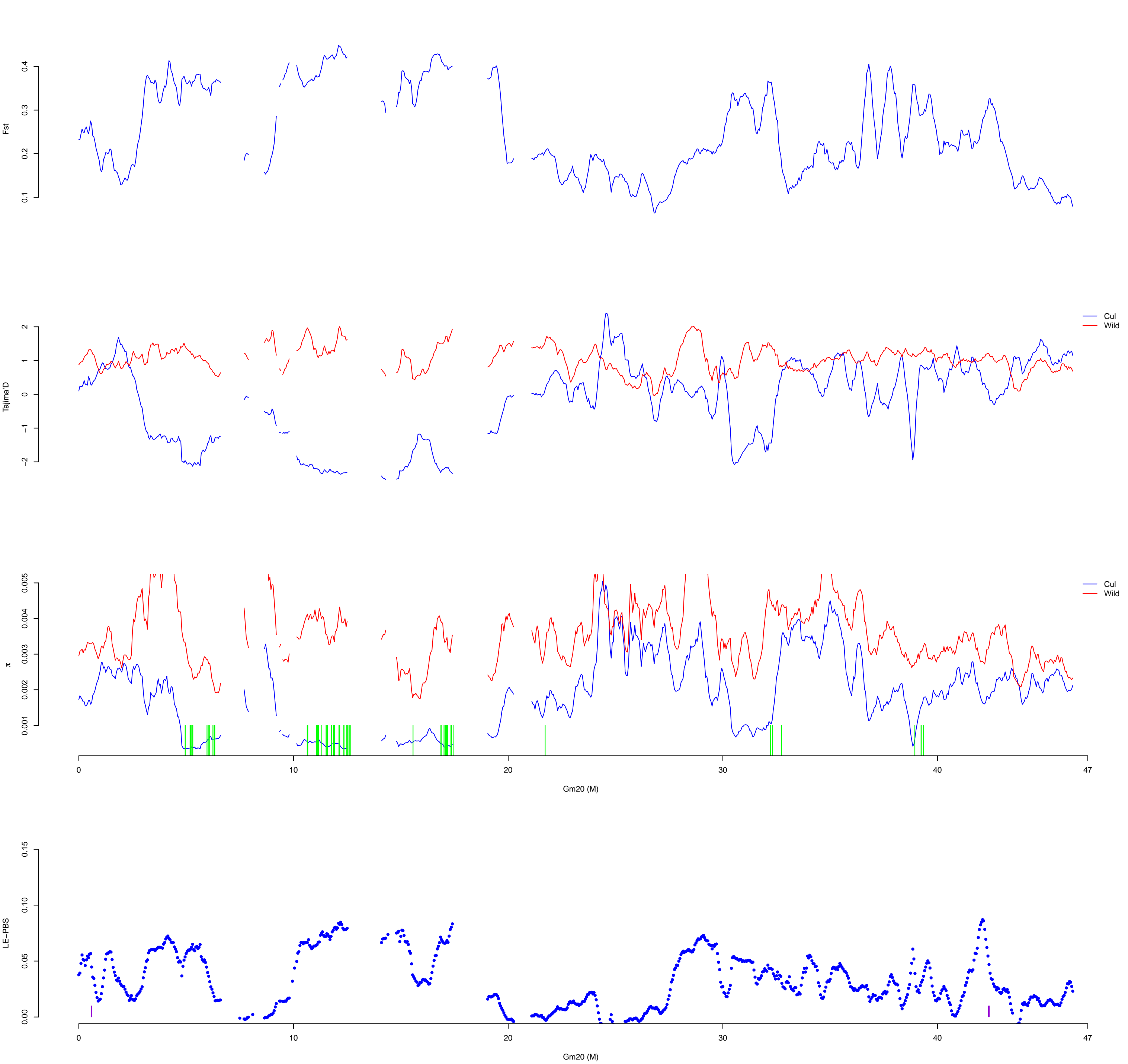

Supplement: Additional file 8 — The diversity pattern of artificial selection regions during domestication and genetic improvement based on Fst, Tajima’s D, π, or PBS’s analysis (between Landraces and elite cultivars). The square frames along the chromosome indicate regions selected during domestication (green) and genetic improvement (purple). [file 1471-2164-14-579-S8.pdf]

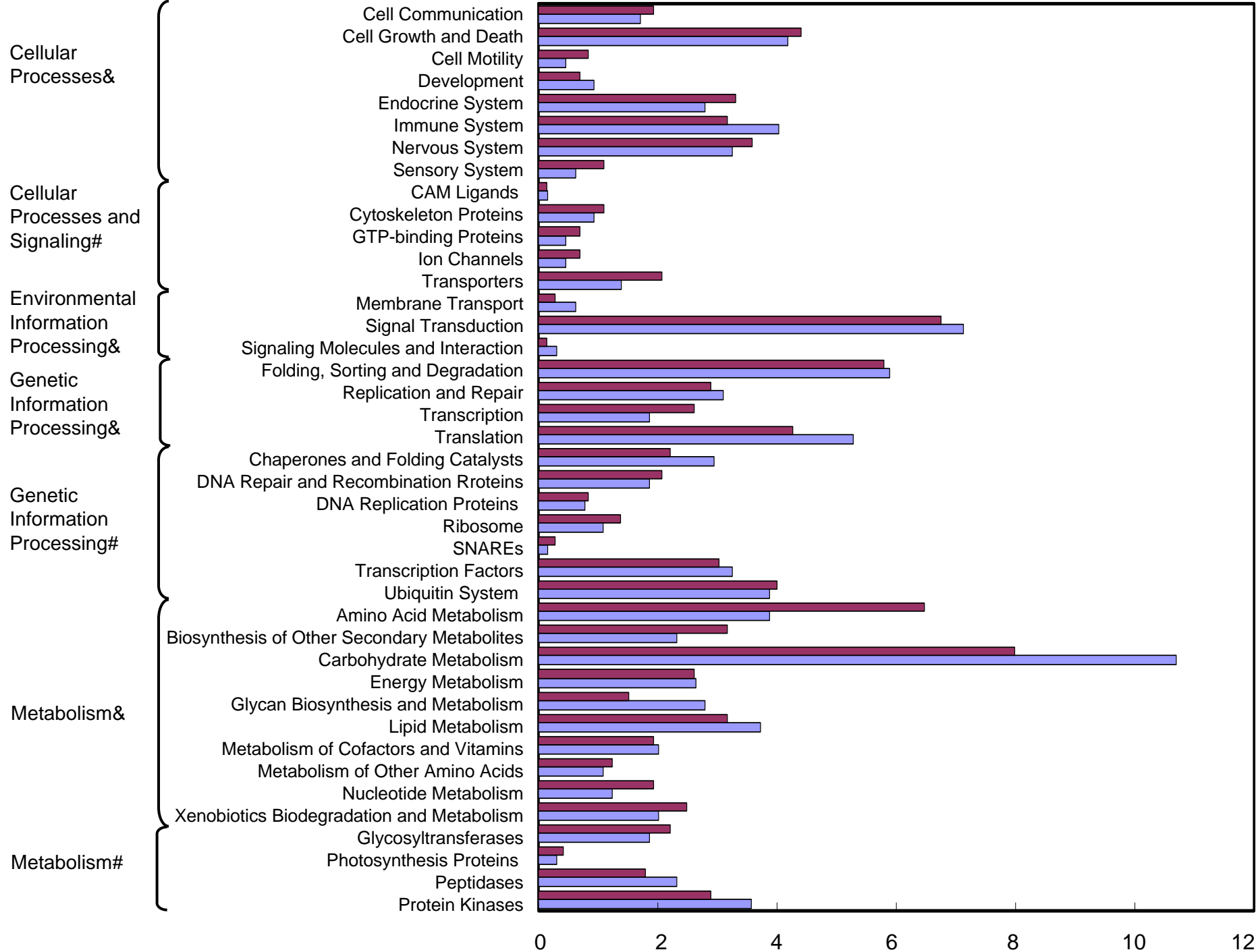

Supplement: Additional file 9 — Pathway analysis for domestication and improvement genes by KEGG. The pathway marked with “&” was deduced from KEGG PATHWAY database and marked with “#” deduced from KEGG BRITE database. [file 1471-2164-14-579-S9.pdf]
