# Supplementary material for: Molecular footprints of domestication and improvement in soybean revealed by whole genome re-sequencing
Source: BMC Genomics. 2013 Aug 28;14:579. doi: 10.1186/1471-2164-14-579 (PMC3844514; doi:10.1186/1471-2164-14-579)
Supplement: Additional file 10 — Domestication regions and genes covered by or near reported QTLs for important domestication traits. [file 1471-2164-14-579-S10.pdf]

Table S5A Domestication regions and genes covered by or near reported QTLs for important agronomic traits

Chromosome	Selection region		Gene ID	Marker/QTL located or near to selection region reported in the previous studies		
	Upper	Down		Marker/QTL*	Traits	Reference
Gm01	51252000	51272000	Glyma01g39290.1	AW781285-Sat 305	Flower color	[1]
Gm01	52280000	52320000	Glyma01g40590.1/ Glyma01g40610.1/ Glyma01g40600.1	Sat 414-Satt129	Flower color	[1]
Gm02	46632000	46656000	Glyma02g41570.1/ Glyma02g41560.1	Satt703-Satt202	Seed hardness	[2]
Gm02	46690000	46728000	Glyma02g41630.1	Satt703-Satt202	Seed hardness	[2]
Gm02	46780000	46808000	Glyma02g41690.1/ Glyma02g41700.1/ Glyma02g41710.1	Satt703-Satt202	Seed hardness	[2]
Gm08	3866000	4018000	Glyma08g05470.1/ Glyma08g05480.1/ Glyma08g05450.1/ Glyma08g05490.1/ Glyma08g05500.1/ Glyma08g05460.1/ Glyma08g05560.1/ Glyma08g05610.1/ Glyma08g05510.1/ Glyma08g05570.1/ Glyma08g05600.1/ Glyma08g05530.1/ Glyma08g05550.1/ Glyma08g05590.1/ Glyma08g05440.1/ Glyma08g05580.1/ Glyma08g05540.1	Satt207-Satt493	Seed-coat color	[1]
Gm08	4022000	4050000	Glyma08g05660.1/ Glyma08g05650.1/ Glyma08g05640.1	Satt207-Satt493	Seed-coat color	[1]
Gm08	4178000	4212000	Glyma08g05880.1/ Glyma08g05870.1/ Glyma08g05890.1	Satt207-Satt493	Seed-coat color	[1]
Gm08	4334000	4408000	Glyma08g06170.1/ Glyma08g06160.1/ Glyma08g06120.1/ Glyma08g06180.1/ Glyma08g06150.1/ Glyma08g06130.1	Satt207-Satt493	Seed-coat color	[1]

Gm11	2190000	2210000	Glyma11g03340.1/ Glyma11g03330.1/ Glyma11g03320.1	BE806308-Sat 272	Flower color	[1]
Gm12	35142000	35162000	Glyma12g31550.1	Satt142	Seed weight	
Gm13	4070000	4102000	Glyma13g03900.1/ Glyma13g03880.1	Satt348-Satt160	Seed-coat color	[1]
Gm17	9750000	9784000	Glyma17g12880.1	Satt002-Satt154	Seed weight	[3]
Gm19	34642000	34666000	Glyma19g27360.1/ Glyma19g27340.1/ Glyma19g27350.1	Satt652-Satt284	Lodging, Plant height	[4]
Gm20	11118000	11178000	Glyma20g08030.1/ Glyma20g08020.1/ Glyma20g08040.1/ Glyma20g08010.1	Satt127	Yield, Maturity time, Plant height, Protein content	[2]
Gm20	11308000	11328000	Glyma20g08140.1	Satt127	Yield, Maturity time, Plant height, Protein content	[2]
Gm20	11508000	11536000	Glyma20g08250.1	Satt127	Yield, Maturity time, Plant height, Protein content	[2]
Gm20	11572000	11592000	Glyma20g08260.1	Satt127	Yield, Maturity time, Plant height, Protein content	[2]
Gm20	12106000	12128000	Glyma20g08580.1/ Glyma20g08590.1	Satt127	Yield, Maturity time, Plant height, Protein content	[2]
Gm20	32304000	32326000	Glyma20g22280.1	Sat 174-ssrpqtl 38	Protein content	[5]
Gm20	32726000	32746000	Glyma20g22830.1	Sat 174-ssrpqtl 38	Protein content	[5]

* Putative QTL identified underling domestication traits in interspecific soybean populations

Table S5B Genetic improvement regions and genes covered by or near reported QTLs for important agronomic traits

Chromosome	Selection region		Gene ID	Marker/QTL located or near to selection region reported in the previous studies		
	Upper	Down		Marker/QTL[&]	Traits	Reference
Gm06	5744000	5780000	Glyma06g07820.1/ Glyma06g07840.1	Satt640-Satt422	Seed weight	[6]
Gm06	5792000	5846000	Glyma06g07920.1/ Glyma06g07900.1/ Glyma06g07890.1	Satt640-Satt422	Seed weight	[6]

Gm06	5966000	6018000	Glyma06g08170.1/ Glyma06g08120.1/ Glyma06g08110.1/ Glyma06g08180.1	Satt640-Satt422	Seed weight	[6]
Gm06	6436000	6458000	Glyma06g08820.1/ Glyma06g08830.1/ Glyma06g08840.1	Satt640-Satt422	Seed weight	[6]
Gm06	6540000	6564000	Glyma06g08970.1/ Glyma06g08980.1	Satt640-Satt422	Seed weight	[6]
Gm06	6594000	6628000	Glyma06g09000.1/ Glyma06g09010.1	Satt640-Satt422	Seed weight	[6]
Gm06	28750000	28778000	Glyma06g30010.1	Satt489-Satt100	Seed hardness	[7]
Gm06	28786000	28810000	Glyma06g30020.1	Satt489-Satt100	Seed hardness	[7]
Gm06	28914000	28940000	Glyma06g30040.1/ Glyma06g30050.1	Satt489-Satt100	Seed hardness	[7]
Gm08	6898000	6938000	Glyma08g09710.1/ Glyma08g09720.1	Sat_215-Sat_409	Pod number	[8]
Gm08	6946000	7074000	Glyma08g09820.1/ Glyma08g09870.1/ Glyma08g09780.1/ Glyma08g09840.1/ Glyma08g09800.1/ Glyma08g09790.1/ Glyma08g09830.1/ Glyma08g09760.1/ Glyma08g09810.1/ Glyma08g09750.1/ Glyma08g09740.1/ Glyma08g09880.1/ Glyma08g09860.1	Sat_215-Sat_409	Pod number	[8]
Gm08	7180000	7348000	Glyma08g10030.1/ Glyma08g10100.1/ Glyma08g10080.1/ Glyma08g10140.1/ Glyma08g10070.1/ Glyma08g10020.1/ Glyma08g10110.1/ Glyma08g10120.1/ Glyma08g10180.1/ Glyma08g10130.1/ Glyma08g10050.1/ Glyma08g10010.1/ Glyma08g10040.1/ Glyma08g10160.1	Sat_215-Sat_409	Pod number	[8]

Gm08	7496000	7538000	Glyma08g10360.1/<br>Glyma08g10370.1/<br>Glyma08g10390.1/<br>Glyma08g10380.1	Sat_215-Sat_409	Pod number	[8]
Gm08	8172000	8286000	Glyma08g11170.1/<br>Glyma08g11200.1/<br>Glyma08g11190.1/<br>Glyma08g11260.1/<br>Glyma08g11310.1/<br>Glyma08g11210.1/<br>Glyma08g11350.1/<br>Glyma08g11240.1/<br>Glyma08g11290.1/<br>Glyma08g11250.1/<br>Glyma08g11180.1/<br>Glyma08g11300.1/<br>Glyma08g11330.1/<br>Glyma08g11280.1/<br>Glyma08g11270.1/<br>Glyma08g11220.1/<br>Glyma08g11340.1/<br>Glyma08g11160.1	Sat_215-Sat_409	Pod number	[8]
Gm08	8292000	8334000	Glyma08g11450.1/<br>Glyma08g11400.1/<br>Glyma08g11380.1/<br>Glyma08g11430.1/<br>Glyma08g11420.1	Sat_215-Sat_409	Pod number	[8]
Gm08	8354000	8466000	Glyma08g11580.1/<br>Glyma08g11610.1/<br>Glyma08g11600.1/<br>Glyma08g11560.1/<br>Glyma08g11530.1/<br>Glyma08g11500.1/<br>Glyma08g11570.1/<br>Glyma08g11520.1/<br>Glyma08g11590.1/<br>Glyma08g11490.1/<br>Glyma08g11510.1/<br>Glyma08g11550.1/<br>Glyma08g11540.1	Sat_215-Sat_409	Pod number	[8]

Gm08	8610000	8648000	Glyma08g11850.1/ Glyma08g11840.1/ Glyma08g11910.1/ Glyma08g11870.1/ Glyma08g11890.1/ Glyma08g11900.1/ Glyma08g11830.1	Sat_215-Sat_409	Pod number	[8]
Gm08	9034000	9088000	Glyma08g12410.1/ Glyma08g12390.1/ Glyma08g12360.1/ Glyma08g12400.1/ Glyma08g12380.1/ Glyma08g12370.1	Sat_215-Sat_409	Pod number	[8]
Gm15	11828000	11848000	Glyma15g15420.1/ Glyma15g15410.1	Sat_124-Satt598	Flower number	[8]
Gm15	11850000	11876000	Glyma15g15440.1/ Glyma15g15430.1	Sat_124-Satt598	Flower number	[8]

[&] Putative QTL identified underling agronomic traits in soybean populations developed using one landrace and one bred cultivar as parents.

Reference

1. Yang K, Jeong N, Moon JK, Lee YH, Lee SH, Kim HM, Hwang CH, Back K, Palmer RG, Jeong SC: **Genetic analysis of genes controlling natural variation of seed coat and flower colors in soybean**. *J Hered* 2010, **101**(6):757-768.
2. Wang D, Graef G, Procopiuk A, Diers B: **Identification of putative QTL that underlie yield in interspecific soybean backcross populations**. *Theoretical and Applied Genetics* 2004, **108**(3):458-467.
3. Liu B, Fujita T, Yan ZH, Sakamoto S, Xu D, Abe J: **QTL mapping of domestication-related traits in soybean (Glycine max)**. *Ann bot* 2007, **100**(5):1027-1038.
4. Li W, Han Y, Zhang D, Yang M, Teng W, Jiang Z, Qiu L, Sun G: **Genetic diversity in soybean genotypes from north-eastern China and identification of candidate markers associated with maturity rating**. *Plant breeding* 2008, **127**(5):494-500.
5. Bolon YT, Joseph B, Cannon S, Graham M, Diers B, Farmer A, May G, Muehlbauer G, Specht J, Tu Z: **Complementary genetic and genomic approaches help characterize the linkage group I seed protein QTL in soybean**. *BMC Plant Biology* 2010, **10**(1):41.
6. Wang X, Xu Y, Li G, Li H, Gen W, Zhang Y: **Mapping quantitative trait loci for 100-Seed weight in soybean (*Glycine max* L. Merr.)**. *Acta Agron Sin* 2010, **36**(10):1674-1682.
7. Watanabe S, Tajuddin T, Yamanaka N, Hayashi M, Harada K: **Analysis of QTLs for reproductive development and seed quality traits in soybean using recombinant inbred lines**. *Breeding Sci* 2004, **54**(4):399-407.
8. Zhang D, Cheng H, Wang H, Zhang H, Liu C, Yu D: **Identification of genomic regions determining flower and pod numbers development in soybean (*Glycine max* L.)**. *J Genet Genomics* 2010, **37**(8):545-556.
